# Single-cell RNA-seq reveals a concomitant delay in differentiation and cell cycle of aged hematopoietic stem cells

**DOI:** 10.1186/s12915-021-00955-z

**Published:** 2021-02-01

**Authors:** Léonard Hérault, Mathilde Poplineau, Adrien Mazuel, Nadine Platet, Élisabeth Remy, Estelle Duprez

**Affiliations:** 1 Epigenetic Factors in Normal and Malignant Hematopoiesis Team, Aix Marseille Université, CNRS, INSERM, Institut Paoli-Calmettes, CRCM, Marseille, France; 2 Aix Marseille Université, CNRS, Centrale Marseille, I2M, Marseille, France

**Keywords:** Single-cell RNA-seq, Hematopoietic stem cell, Aging, Trajectories, Differentiation, Cell cycle

## Abstract

**Background:**

Hematopoietic stem cells (HSCs) are the guarantor of the proper functioning of hematopoiesis due to their incredible diversity of potential. During aging, heterogeneity of HSCs changes, contributing to the deterioration of the immune system. In this study, we revisited mouse HSC compartment and its transcriptional plasticity during aging at unicellular scale.

**Results:**

Through the analysis of 15,000 young and aged transcriptomes, we identified 15 groups of HSCs revealing rare and new specific HSC abilities that change with age. The implantation of new trajectories complemented with the analysis of transcription factor activities pointed consecutive states of HSC differentiation that were delayed by aging and explained the bias in differentiation of older HSCs. Moreover, reassigning cell cycle phases for each HSC clearly highlighted an imbalance of the cell cycle regulators of very immature aged HSCs that may contribute to their accumulation in an undifferentiated state.

**Conclusions:**

Our results establish a new reference map of HSC differentiation in young and aged mice and reveal a potential mechanism that delays the differentiation of aged HSCs and could promote the emergence of age-related hematologic diseases.

**Supplementary Information:**

The online version contains supplementary material available at 10.1186/s12915-021-00955-z.

## Background

The hematopoietic stem cell (HSC) is an adult tissue stem cell residing in the bone marrow (BM), with multipotent differentiation, regenerative and self-renewal abilities, the proper functioning of which is a guarantee of a healthy immune system. HSC properties have been extensively studied thanks to the use of specific surface markers and multicolored fluorescence-assisted cell sorting (FACS) analyses that have made it possible to isolate them and test their properties during serial grafts [1, 2]. This cell-surface marker-based HSC characterization has shaped the classical but largely revisited hematopoietic model, in which the long-term HSC (LTHSC), at the top of the hierarchy, undergoes a lineage commitment through a series of discrete intermediate progenitor stages in a stepwise manner. This approach has helped to categorize short-term HSC (STHSC) and multipotent progenitor populations (MPP2, MPP3, and MPP4) [3–5].

HSCs are not a homogeneous cell population and each HSC does not contribute equivalently to all blood lineages. HSC heterogeneity was first suggested with single cell transplantation experiments showing that phenotypically identical HSC differs in self-renewal abilities and lineage differentiation potential [6–8]. Next, single cell transcriptomic approaches combined with lineage tracing suggested an initiation of transcriptional lineage programs in HSCs, which bias their differentiation potential [9, 10] supporting an early HSC lineage segregation and a continuous differentiation model [11]. Thus, it is now admitted that each individual HSC, although sharing the same marker combination, differs in terms of functional outputs and molecular signature [12–14].

This HSC heterogeneity has physiological consequences upon aging. Hematopoietic aging is associated with a reduced production of red blood cells and lymphocytes concomitant to an increase of myeloid and megakaryocytic cells that promote immunosenescence and myeloid malignancies [15, 16]. Evidence indicates that these alterations of the hematopoietic system come from an age-related modification of the HSC compartment. Intrinsic changes such as accumulation of DNA damage, changes in the activity of epigenetic modulators, and imbalance between repressive and activating histone marks in HSCs have emerged as contributing factors of hematopoiesis aging [17, 18]. HSCs that are heterogeneous with respect to their self-renewal and differentiation capacities at birth pass through clonal selection over time due to environmental cues [19]. This results in an increase in myeloid- and megakaryocytic-biased HSCs within the phenotypic LTHSC compartment [20, 21]. Thus, aging is not only reflecting an intrinsic uniform change in lineage output of the HSCs but is rather due to a shift in the relative proportion of HSCs with different characteristics [22].

Previous studies on age-related transcriptomic changes of HSCs at the single cell resolution have revealed an expansion of platelet-primed HSCs [23] and a gain of a self-renewal expression program [24] with aging. However, the resolution of the analyses in particular regarding the proportion of the different HSC populations and their variation upon aging were limited due to the small number of analyzed cells and sorting strategies. Here, we took advantage of the 10x Genomics approaches and the development of new bioinformatic methods and tools to increase the resolution and revisit the transcriptional heterogeneity and change upon aging of the HSC compartment. By analyzing 15,000 single murine hematopoietic stem and progenitor cells (HSPC) transcriptomes, we detected new rare HSC subpopulations that accumulate upon aging. We also highlighted transcriptional program changes linked to cell cycle activity during aging that participate to the HSC age-related alterations.

## Results

### Stratification of HSPCs using single-cell transcriptome analysis highlighted 15 different clusters

**Fig. 1:**
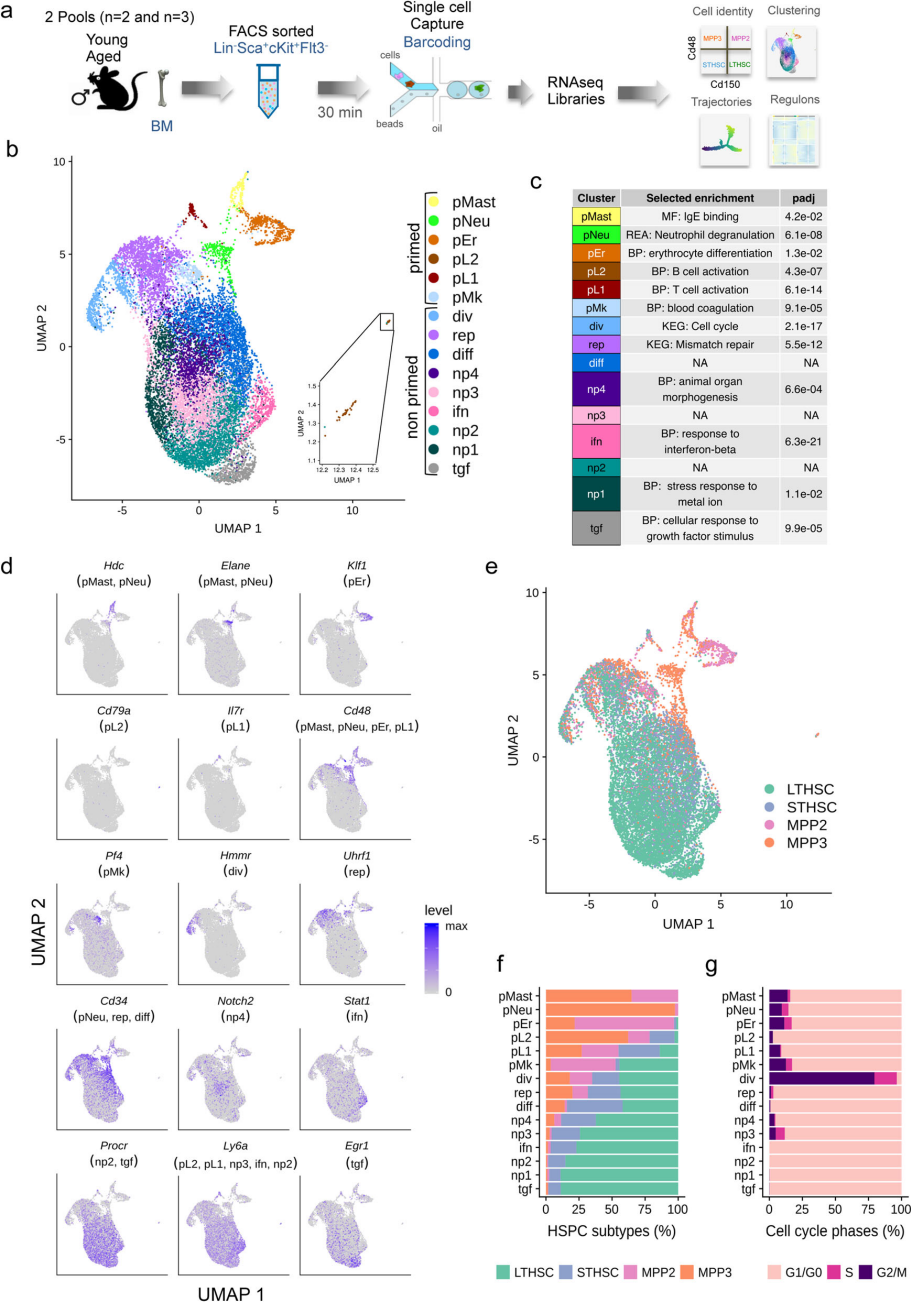
Unsupervised clustering of young and aged HSPCs revealed 15 clusters gathering mainly immature and to a lesser extend lineage-primed HSPCs. **a** Overview of the scRNA-seq sample preparation and analysis. Cells were isolated from bone marrow (BM) of young and aged mice and pooled to obtain 2 pools for each age. Pools of 2 and 3 BMs for both young and aged mice were analyzed. BM cells were FACS sorted to purify Lin^−^, Sca-1^+^, c-Kit^+^ (LSK) Flt3^−^ cells that defined the HSPCs. The four pools of HSPCs were processed using droplet-based single cell sequencing (10X Genomics) and multiple analyses were performed using bioinformatics tools to characterize aging effects. **b** UMAP plot of young and aged HSPCs (15,000 cells) analyzed using Seurat. Colors marked the 15 distinct clusters identified by unsupervised clustering and characterized with differential gene expression and gene set enrichment analyses. Each dot represents a cell. **c** Selected enrichment of our analysis (Gene Ontology, KEG and Reactome pathways Supplemental Table S3) for markers of each cluster and corresponding *p* values adjusted for multiple testing (padj). NA indicates non-relevant enrichment. **d** UMAP plots colored by expression of selected cluster markers. Cluster names are indicated in parenthesis. **e** Localization in the UMAP of LTHSCs, STHSCs, MPP2 and MPP3, identified by supervised classification. **f** Percentage of LTHSCs, STHSCs, MPP2 and MPP3 within the HSPC population, in each of the 15 clusters. **g** Percentage of computationally assigned cell cycle (G1/G0, S and G2/M) phases in each of the 15 clusters

To characterize HSC populations by single cell RNAseq (scRNA-seq), we purified HSPCs, including LTHSCs, STHSCs, MPP2, and MPP3 by FACS from BM pools of young (*n* = 5; 2–3 months) and aged (*n* = 5; 17–18 months) mice applying the widely used Lin^−^, Sca1^+^, cKit^+^ (LSK) marker strategy with the addition of the Flt3 marker to exclude the Flt3^+^ LSKs also referenced as MPP4 (Fig. 1a and Additional file 1: Fig. S1A). Four pools (2 pools of young and 2 of aged) of thousands HSPCs were subjected to 10x Genomics Chromium capture platform and a total of 15,000 single HSPC transcriptomes were sequenced (young pools, with 5189 and 2244 cells and aged pools with 3328 and 4154 cells after quality control; Additional file 2: Table S1). As we made the assumption that aging would not dramatically modify HSC identity, we first analyzed young and aged HSPCs together using Seurat workflow [25] for the integration of the different samples to correct batch effect. Reduction of dimension and unsupervised clustering were performed on cell-cycle-corrected data using Uniform Manifold Approximation and Projection (UMAP) [26]. A total of 15 clusters were identified (Fig. 1b), which were characterized further by identifying their markers using differential expressed gene (DEG) analysis on the log-normalized data without any correction (Additional file 3: Table S2) and by deducing their characteristics (Fig. 1c) from gene set enrichment analysis (Gene Ontology, KEG, and Reactome pathways; Additional file 4: Table S3) and gene signatures related to hematopoiesis (Additional file 5: Table S4a). Six clusters were classified as lineage-primed clusters as they were clearly enriched for HSPCs with megakaryocyte (pMk), erythroid (pEr), neutrophil (pNeu), mastocyte (pMast), and lymphocyte (pL1 and pL2) commitment gene markers (Fig. 1b–d; Additional file 3: Table S2, Additional file 4: Table S3 and Additional file 5: Table S4a). Nine clusters were considered as non-primed due to their lack in expression of lineage restricted-genes. They accounted for a large majority of the analyzed cells (90%) (Fig. 1b–d; Additional file 3: Tables S2 and Additional file 5: S4b). The 4 phenotypically distinct HSPCs, LTHSCs, STHSCs, MPP2, and MPP3 were assigned by supervised classification using previously published scRNA-seq data of FACS-labeled HSPCs [11] (Additional file 1: Fig. S1B) and were superimposed on the UMAP (Fig. 1e). This showed that globally MPP2 and MPP3 were composed of lineage-primed clusters, suggesting their “more differentiated” state in comparison to the remaining clusters while LTHSCs were enriched with non-primed clusters (Fig. 1e). Distribution of the different populations among the clusters showed that the neutrophil-biased cluster (pNeu) was almost exclusively enriched with MPP3 (98%), while pMast and pEr were enriched with both MPP2 and MPP3 (Fig. 1f and Additional file 5: Table S4c). The pMK cluster was composed of almost 50% of LTHSCs, supporting previous work suggesting that platelet-biased stem cells reside at the apex of the HSC hierarchy [27].

Four non-primed clusters, np1, np2, np3, and np4, were overlapping and positioned at the center of the UMAP (Fig. 1b) with few specific gene markers (Fig. 1c, d; Additional file 3: Table S2 and Additional file 1: Fig. S2). They were characterized by a high percentage of LTHSCs (Fig. 1e, f; Additional file 3: Table S2). By contrast, 2 clusters, also composed mainly of LTHSCs, harbored a very distinguishable signature for growth factor signaling (tgf) and interferon response (ifn) respectively (Fig. 1b–d and Additional file 4: Table S3), witnessing the existence of cells with signaling features at the top of the differentiation hierarchy. The remaining 3 clusters (diff, div and rep) were composed of less than 50% of LTHSCs (Fig. 1e and Additional file 5: Table S4c) suggesting their intermediate state in term of differentiation. The cluster named diff had very few distinguishable markers but was enriched with *Cd34* expressing cells (Fig. 1d and Additional file 1: Fig. S2). Interestingly, this cluster was the most enriched with STHSCs (Fig. 1e and Supplemental Table S4c), which have been characterized by the appearance of the Cd34 at their surface [2]. The div cluster, characterized by enrichment for the cell cycle KEGG pathway (Fig. 1c and Additional file 4: Table S3) and genes involved in division such as *Hmmr2* (Fig. 1d and Additional file 1: Fig. S2), was particularly different from the other clusters by its enrichment in G2/M cells (Fig. 1g and Additional file 5: Table S4d). The rep cluster was characterized by genes involved in DNA repair and replication and presented a specific high expression of *Uhfr* (Fig. 1c, d and Additional file 1: Fig. S2 and Additional file 4: Table S3).

As a whole, these results highlight the interest of gene expression signature to identify heterogeneity in the HSC population. They support the presence of differentiation-biased cells in the immature hematopoietic compartment and demonstrate that transcriptional programs can subdivide HSPCs in different clusters besides their classical differentiation state defined by cell surface markers.

### Aging affects HSPC clusters differently

**Fig. 2:**
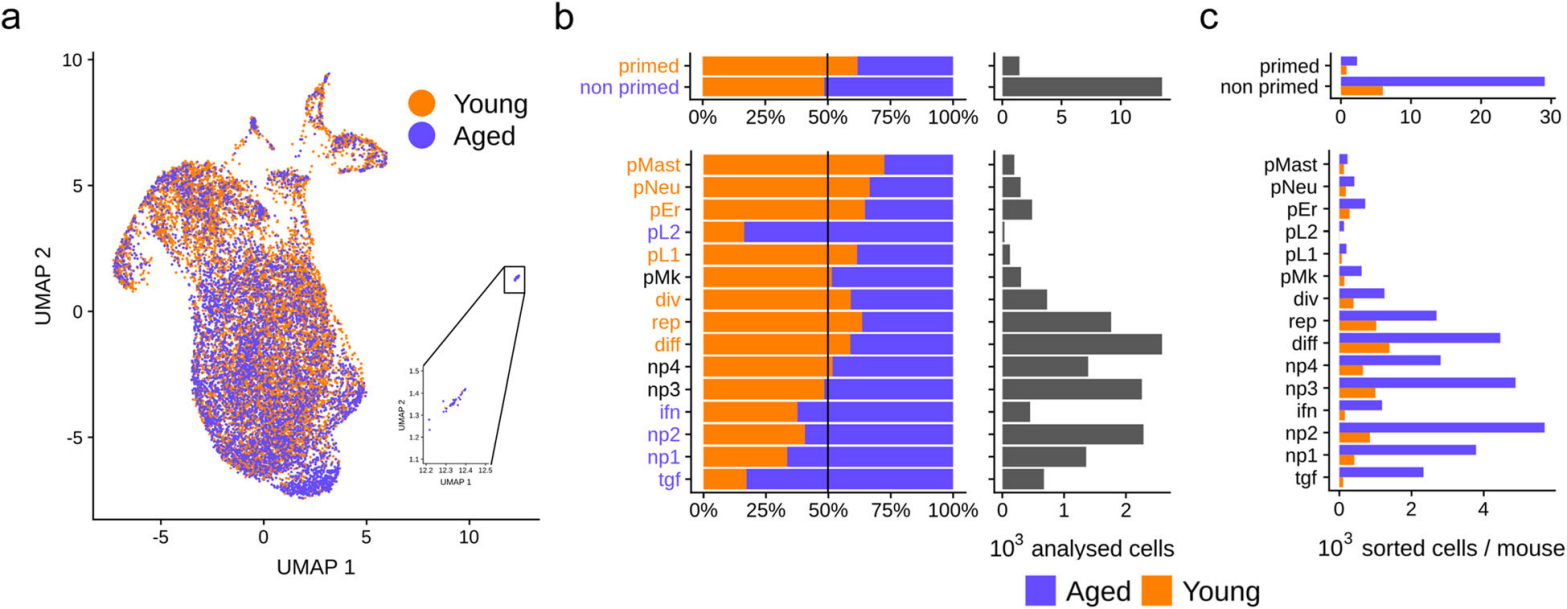
Aging affects HSPC clusters differently. **a** UMAP plot (same as in Fig. 1b) showing the young (orange) and the aged (purple) HSPCs. **b** Distribution of young (orange) and aged (purple) HSPCs in the clusters. Left panel presents percentages of young and aged HSPCs in primed/non-primed clusters gathered and in each of the 15 clusters. The black vertical line indicates expected young and aged cell proportions according to dataset size. Names of the clusters for which proportion of aged or young cells was significantly higher than expected (hypergeometric test *p* value < 0.05) are colored in purple for aged cells and in orange for young cells. On the right, barplots represent the number of cells composing each ensemble: primed/non primed clusters gathered and individual clusters. **c** Absolute number per mouse of young and aged sorted HSPCs into the 15 groups

To assess the aging effect at the level of HSC populations, we first confirmed by FACS analyses and by transcriptomic-based cell population predictions the well-described accumulation of LTHSCs that occurs at the expense of the STHSCs and the MPP3 upon aging (Additional file 1: Fig. S1C and D). Analysis of young versus aged cells in the UMAP plot showed that aged cells were significantly more distributed in the non-primed clusters while lineage-primed clusters were enriched with young HSPCs (Fig. 2a, b). Indeed, the primed lymphoid (pL1) and the myeloid primed (pMast, pNeu, and pEr) clusters were predominantly composed of young cells (Fig. 2b and Additional file 5: Table S4e). An exception was observed for the pL2 cluster; although representing very few cells, this cluster, characterized with B cell markers, was comprised mainly of aged ones (Fig. 2b and Additional file 5: Table S4b and e). Interestingly, these potentially aged B-biased cells were characterized, in addition to the expression of early B cell markers such as *Ly6d* and *Cd79a* (Fig. 1d, Additional file 1: Fig. S3 and Additional file 3: Table S2), by *Trp53inp1* expression, for which we recently showed its involvement in the blockage of early B cell developmental step [28]. Analysis of absolute numbers of sorted HSPCs per mouse confirmed the higher increase of the non-primed clusters in comparison to the primed ones upon aging (Fig. 2c). This increase in HSC subpopulations was specially marked for the non-primed np1, np2, ifn, and tgf clusters that were largely amplified in aged condition (Fig. 2c and Additional file 5: Table S4e). This result highlights an amplification of LTHSCs being able to respond to different stimuli such as inf and tgf signaling that may overlap with previously reported HSC sub-populations, which promotes differential responses to inflammatory challenge in aged HSCs [29]. Noticeably, we observed that the age-induced decrease of pL1 cluster and increase of tgf cluster were mainly driven by one batch, specific for each of them (Additional file 5: Table S4e) witnessing heterogeneity of aging inter mouse groups.

From these results, we conclude that neither HSPCs nor individuals are affected equally by aging. Globally, aged hematopoiesis is stemming from HSPCs that are not lineage primed and but characterized by specific signaling signatures.

### Gene expression is more altered upon aging in non-primed clusters, with a loss of differentiation and a gain of hemostasis signatures

To reveal age-dependent changes in gene expression, we first compared the transcriptomes of young and aged HSPCs. Differentially expressed gene (DEG) analysis highlighted a global HSC aging signature that was characterized by an upregulation of the stress gene *Nupr1*, the platelet-lineage markers *Vwf* and *Clu*, and markers of undifferentiated HSPCs such as *Procr* and *Slamf1*, as well as by a downregulation of genes that mark HSC differentiation, such as *Cd34* and *Cd48* (Additional file 1: Fig. S4 and Additional file 6: Table S5). These results are in line with the altered differentiation potential and platelet bias of aged HSPCs [23] and a recently published comprehensive HSC aging signature [30].

**Fig. 3:**
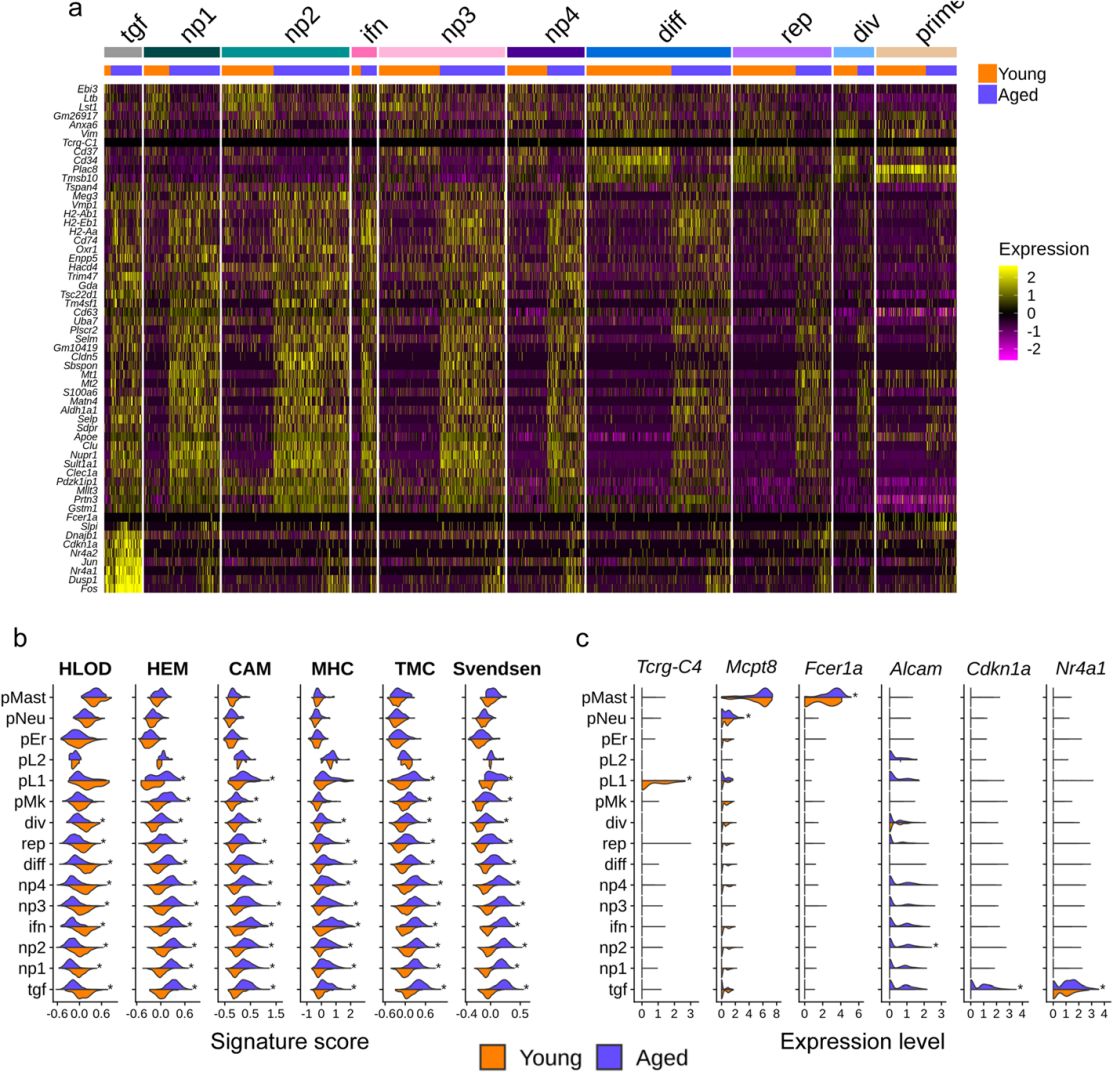
Gene expression is more impaired during aging in non-primed clusters with loss of differentiation and gain of hemostasis signatures.** a** Heatmap of the most significant differentially expressed genes (DEGs) during aging (*p* value < 0.05 and log fold change > 0.5 in at least one cluster) in the different clusters revealed by Seurat analysis (Fig. 1b). The lineage-primed clusters are gathered and labeled as primed. The upper colored bars indicate cluster identity according to the color code in Fig. 1b. The lower colored bars indicate the proportion of young (orange) and aged (purple) cells in a given cluster. Gene expression are standardized across the entire dataset. **b** Combined violin plots showing signature scores (x-axis) in young (orange) and aged (purple) conditions per cluster. Signature scores represent the global expression of annotated genes for selected terms from enrichment analysis issued from DEGs during aging (*p* value < 0.05 and log fold change > 0.25 in at least one cluster) and for the HSC aging signature of Svendsen et al. (significant enrichment, hypergeometric test *p* value < 10^−78^). Significant terms (enrichment gprofiler *p* value < 0.05) are: Hematopoietic or Lymphoid Organ Development (HLOD) retrieved from GO:Biological Process, Hemostasis (HEM) retrieved from REACTOM pathways, Cell Adhesion Molecule (CAM) retrieved from KEGG pathways, MHC protein complex (MHC) retrieved from GO:Cellular Component, Transcriptional Miss-regulation in Cancer (TMC) retrieved from KEGG pathway. See supplementary Tables 7A & B for the lists of genes enriched in the terms. Stars show significant differences between the signature scores of young and aged cells, per cluster (average score difference > 0.1 and *p* value < 0.05). **c** Combined violin plot showing aging marker expression in young (orange) and aged (purple) conditions for the different clusters. Stars show significant differences of gene expression between young and aged cells (average log fold change > 0.25 and *p* value < 0.05)

In order to assess the heterogeneity of transcriptome changes upon aging according to HSC clusters, we analyzed changes in gene expression of each cluster separately (Additional file 7: Table S6). Heatmap of the most differentially expressed genes (DEGs) (log fold change > 0.5) upon aging analyzed per clusters showed that the non-primed clusters exhibited more differences in their transcriptome than the primed ones and that these differences were towards an increase of gene expression rather than a decrease, suggesting an increased cell-to-cell transcriptional variability upon aging (Fig. 3a and Additional file 1: Fig. S6). For these non-primed clusters, except for the tgf cluster, the differential gene expression analysis per cluster followed the aging signature that was observed when analyzing the totality of the cells (*R*^2^ > 0.8; Additional file 6: Fig. S5). Enrichment analysis of DEGs upon aging revealed a negative regulation of hematopoietic or lymphoid organ development (HLOD) marked by the downregulation of *Cd34*, *Plac8*, and *Foxo3* (Additional file 8: Table S7A), together with a positive regulation of hemostasis with *Clu* and *Selp* increased expression, Cell Adhesions Molecule (CAM) genes such as *Alcam*, *Jam2*, Major Histocompatibility Complex (MHC) H-2 genes and genes involved in transcriptional miss-regulation in cancer (TMC) (Additional file 8: Table S7B). TMC enrichment, in addition to TFs such as *Fli1* and *Pbx1*, relies on cell cycle kinase inhibitors *Cdkn1a* and *Cdkn2c* and the stress response gene *Nupr1* suggesting a deregulation of the cell cycle phases upon aging. Globally, we found that aging feature-score differences were more pronounced in the non-primed clusters than in the lineage-primed ones (Fig. 3b; Additional file 8: Table S7C). However, we highlighted that two lineage-primed clusters, the pL1 and pMK clusters, were transcriptionally affected by aging, with an increase in HEM, TMC, and CAM signatures (Fig. 3b and Additional file 8: Table S7C). These observations were confirmed by analyzing a computed aging signature score retrieved from a comprehensive aging signature [30] across the clusters (Fig. 3b and Additional file 8: Table S7C). Looking at some genes individually, we were able to highlight some age-related changes affecting particular clusters. We observed a downregulation of the T cell gene *Tcrg-C4* in the aged pL1 cluster and an upregulation of the protease mast cell gene *Mcpt8* and myeloid integrin gene *Fcer1a* in aged pMast and pNeu cluster respectively (Fig. 3c and Additional file 7: Table S6). We observed an upregulation of *Alcam* required for HSC maintenance in aged np2 cluster (Fig. 3c and Additional file 7: Table S6). Finally, we also observed a very specific transcriptome in aged tgf cluster characterized by an increase of genes involved in HSC quiescence such as *Cdkn1a*, *Nr4a1* (Fig. 3c), which were clustered together in the heatmap of DEGs upon aging (Fig. 3a).

Altogether, our results revealed particular age-related changes mostly affecting the transcriptome of HSPCs from non-primed clusters and characterized by a loss of differentiation genes that could account for the functional changes of the aged hematopoietic compartment.

### Differentiation trajectory shows a HSPC progression towards T, Mast/Neu and Mk/Er fates that is altered with age

**Fig. 4:**
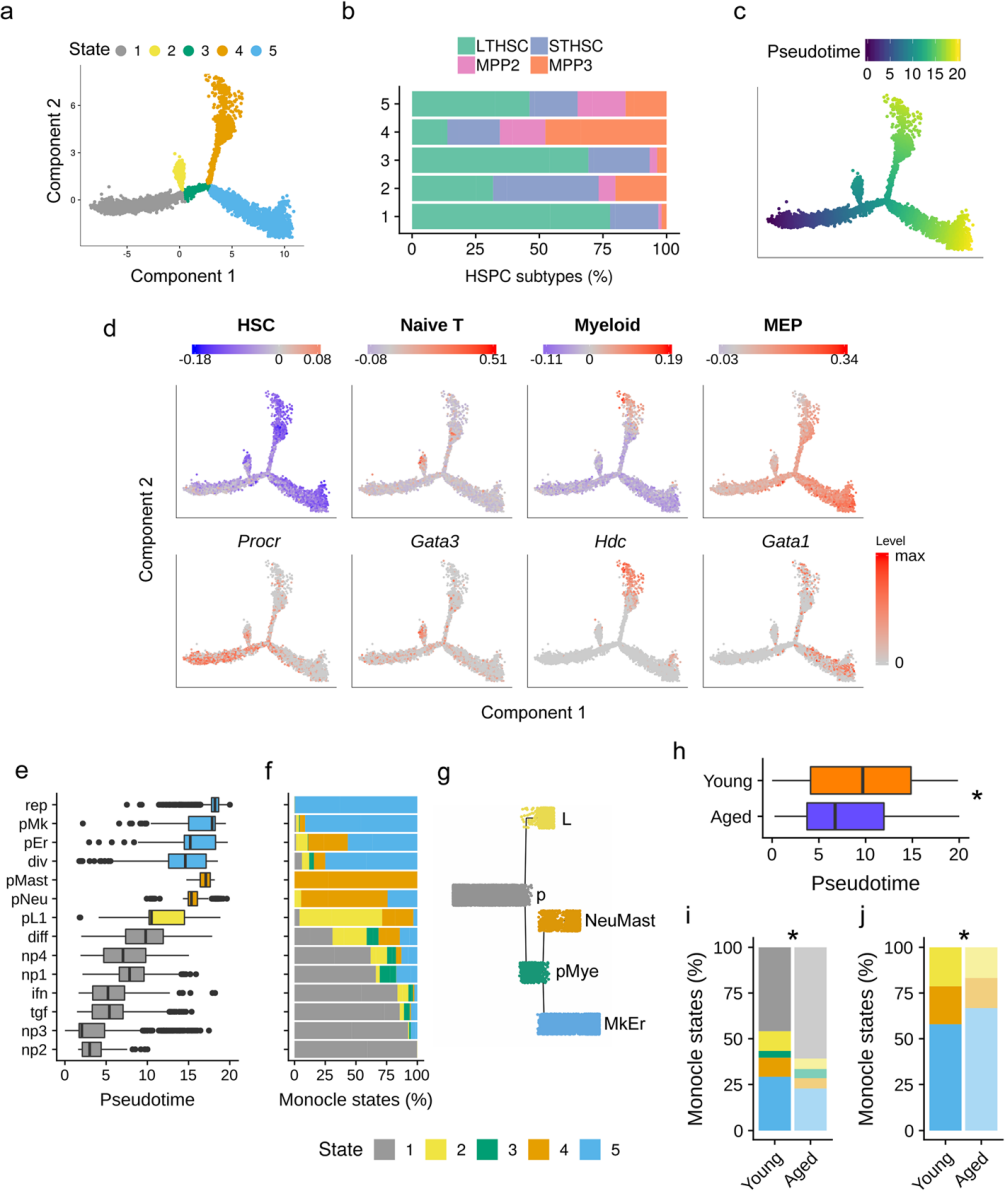
HSPC differentiation trajectory revealed a clear split between L, MastNeu and MkEr primed cells. **a** Differentiation trajectory generated using Monocle 2 with all HSPCs excepted pL2 cells that were excluded. Cells are colored according to five states (1 gray, 2 yellow, 3 green, 4 orange and 5 blue), which partition the trajectory. **b** Barplots representing the LTHSC, STHSC, MPP2 and MPP3 proportions in the five states. **c** HSPC differentiation trajectory colored according to HSPC pseudotime values and representing their differentiation progression. **d** HSPC differentiation trajectories colored according to HSPC scores for hematopoietic lineage signatures retrieved from the literature (upper panel) and according to the expression level of HSPC differentiation markers (lower panel). For signatures, positive (red) or negative (blue) scores indicate whether the expression of the tested gene set is more or less important than an equivalent control gene set. Signatures identified are HSC, naïve T, Myeloid and MEP (Megakaryocyte Erythrocyte Progenitor). HSPC differentiation markers shown are: *Procr* for LTHSCs, *Gata3* for lymphoid-primed HSPCs, *Hdc* for myeloid-primed HSPCs and *Gata1* for erythrocyte-megakaryocyte-primed HSPCs**. e** Repartition of the Seurat clusters along the pseudotime. Box plots (medians) of pseudotime values are colored according to the most represented state. **f** Repartition (in percentage) of the different states (1 to 5) of the trajectory for each Seurat cluster. **g** Tree representation of HSC differentiation trajectory, edges representing the states (state 1 in gray, 2 yellow, 3 green, 4 orange, and 5 blue), and nodes standing for pseudotime points: the starting point (s), the first bifurcation point (p), the primed Myeloid bifurcation point (pMye); and the three fates Lymphocyte (L), Neutrophil/Mastocyte (NeuMast) and Megakaryocyte/Erythroid (MkEr). **h** Boxplot of pseudotime values for young and aged cells. * indicates a significant difference between young and aged pseudotime value distribution (median difference > 2.9, *p* value < 10^−16^ Wilcoxon rank sum test) **i**, **j** Percentage of Monocle states in young and aged conditions, when considering all states (**i**) or only states 2, 4, and 5 (**j**). * indicates a significant dependence between state and age repartitions (*p* value < 10^−10^ Pearson’s chi-squared test)

It has been recently suggested that HSCs undergo a continuous differentiation process rather than a stepwise process [12]. In order to better capture and understand the progression of this differentiation process during aging, we constructed pseudotime trajectories by ordering HSPCs based on the similarities between their expression profiles with Monocle [31]. We first generated the trajectories of young and aged HSPCs separately, which showed a very similar shape, with the exception of a group of cells standing apart from the aged trajectory, and made exclusively of pL2 cells (Additional file 1: Fig. S7A). Because these cells were detected only in aged HSPCs and were clearly distant from the rest of the cells in the UMAP (Fig. 1b), we excluded them for cell pooling and ordering for both ages. Thus, we analyzed the differentiation trajectory inferred from young and aged cells pooled together, without pL2 cells. The resulting trajectory was partitioned into 5 segments, called Monocle states 1, 2, 3, 4, and 5 (Fig. 4a). The departure of the trajectory was identified at the extremity of the state 1, as this state possessed the highest percentage of LTHSCs (Fig. 4b, c). States 2, 4, and 5 were enriched with MPPs suggesting their progression towards differentiated states (Fig. 4b, c). We characterized the 5 states of the trajectory with previously published signatures related to HSPCs and hematopoiesis (referenced in Additional file 9: Table S8A) and with our state marker analysis (Additional file 9: Table S8B & S8C). We revealed that HSPCs in state 1 expressed a HSC signature with especially cells expressing the dormant HSC marker (*Procr*); state 2 cells (after the first bifurcation) were characterized with naive T cell signature and were expressing *Gata3*, suggesting a primed-lymphoid differentiation state (Fig. 4d); state 4 cells were characterized by a myeloid signature [32] and high expression of *Hdc*, previously reported as a marker of myeloid biased HSPCs [33], while cells in state 5 presented a Megakaryocyte Erythrocyte Progenitors (MEP) signature [34] and expressed *Gata1* a MEP marker (Fig. 4d).

Analysis of Seurat cluster position and spreading on the trajectory strengthened the pseudotime differentiation relevance with lineage-primed clusters located at the two extremities of the trajectory and suggested a differentiation specificity of the states (Fig. 4e; Additional file 1: Fig. S8). In addition, we assessed the similarity of our pseudotime cell values with a published HSC score to validate their degree of stemness [35]. By doing so, we identified the two non-primed clusters np2 and np3, located at the beginning of the trajectory, as the most immature ones (Additional file 1: Fig. S9). Analysis of the five state proportions across the clusters revealed a first bifurcation separating pL1 cells (state 2), from cells primed for myeloid lineages (state 3), and then a clear branching between Neu/Mast-primed (NeuMast) HSPCs (state 4) and Mk/Er-primed (MkEr) HSPCs (state 5) (Fig. 4f). The specificity of state 5 for megakaryocyte differentiation was supported by the high representation of the rep cluster (Fig. 4f), characterized by a reparation gene signature (Additional file 4: Table S3), which was previously associated with megakaryocyte fate [36]. Separate pseudotime ordering of young and aged HSPCs provided very similar segregation between the lineage-primed HSPCs, with one bifurcation from LTHSC (state 6) towards Neu/Mast-primed (NeuMast) HSPCs (state 7) and Mk/Er-primed (MkEr) HSPCs (state 8) (Additional file 1: Fig. S7A-E). However, the bifurcation towards the lymphocyte fate was not retrieved probably because of the reduction of the pL1 cell number due to the sample splitting (Additional file 1: Fig. S7A). Hence, to synthetize our analyses, we proposed a tree-representation of the HSC differentiation trajectory (Fig. 4g) where nodes stand for pseudotime points, and edges for Monocles states. It contains 6 nodes: a root, the starting point (s); two internal nodes, the first bifurcation point (p) and primed Myeloid bifurcation point (pMye); and three leave nodes, the three fates Lymphoid (L), Neutrophils/Mastocytes (NeuMast), and Megakaryocyte/Erythroid (MkEr).

Next, we compared the differentiation progression of young and aged HSPCs. Aged HSPCs appear to be significantly delayed in the pseudotime (Fig. 4h) while Seurat cluster spreading along the trajectory showed no clear differences of any cluster pseudotime position according to age (no median difference higher than 0.8 unit of pseudotime; Additional file 1: Fig. S10A). Looking at the proportion of the different Monocle states of the trajectory according to age revealed an increase in aged HSPCs in states 1 and 3 in comparison to young ones (Fig. 4i), belonging to the non-primed clusters np3, tgf, ifn, np4, diff, and div (Additional file 1: Fig. S10B). When focusing on cells from states 2, 4, and 5, which reflect the 3 lineage-primed HSPC states, we observed that the proportion of state 5 (MkEr fate) was larger in aged than young condition (Fig. 4j), although age was not affecting the percentage of the Monocle states from lineage-primed cluster cells (Additional file 1: Fig. S10B). This suggests that while less aged HSCs were detected in the three differentiation paths, cells with MkEr fate are more maintained upon aging than the ones towards NeuMast and L fates.

In conclusion, our trajectory analysis revealed a priming of HSPCs for lymphoid lineage that occurs early in the differentiation process and evidenced a clear split between the NeuMast and the MkEr HSC fate identifying an early lineage specification of HSCs (Fig. 4g). While the global shape of the trajectory and the lineage specification of the HSPCs are conserved upon aging, repartition of the aged HSPCs along the differentiation trajectory is altered with a decrease in terminal states 2 and 4 conducing respectively to L and NeuMast fates, in favor to cells of the initial states 1 and 3.

### HSPC differentiation trajectory is associated with transcriptional programs that are altered upon aging

**Fig. 5:**
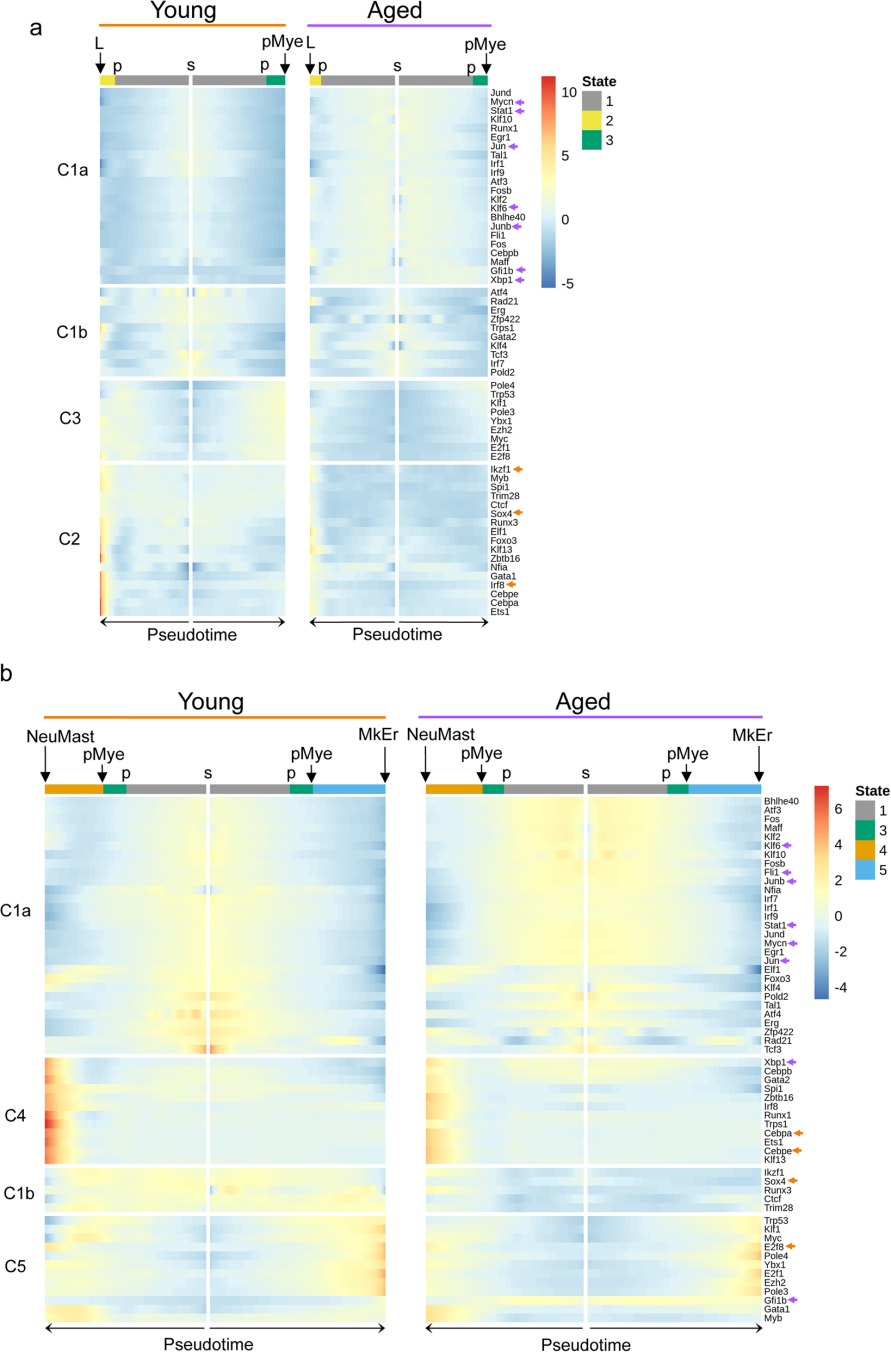
HSPC differentiation trajectory associates with transcriptional programs that are altered upon aging. **a**, **b** Heatmaps showing standardized regulon activity scores, recovered with the AUCell procedure of Scenic, for young (left panel) and aged (right panel) HSPCs across Monocle states. Cells (columns) were ordered according to their pseudotime, and color bars at the top of the heatmaps indicate the state at which cell belongs (1 gray, 2 yellow, 3 green, 4 orange, and 5 blue). Regulons (rows) were hierarchically clustered, based on their activity score in young HSPCs. In **a**, 4 clusters of regulons are highlighted when analyzing regulon activity along pseudotime trajectories from s to L fate and from s to pMye bifurcation point (i.e., across Monocle states 1, 2 and 1, 3). In **b**, regulon activity along pseudotime trajectories from s to NeuMast and from s to MkEr fates (i.e., across states 1, 3, 4 and 1, 3, 5) is analyzed and 4 other clusters of regulons are highlighted. Arrows mark regulons for which a significant difference of activity with aging (average AUCell score difference between young and aged cells > 0.002 and *p* value < 0.05) were found in at least one of the considered states (i.e., states 1, 2, and 3 in **a** and 1, 3, 4, and 5 in **b**). The color indicates if regulon activity is increased (purple) or decreased (orange) in aged condition

Cell fate decision and proper function of HSCs rely on tightly controlled transcriptional programs orchestrated by transcription factor (TF) activity [37]. Since level of the expression of TFs is not sufficient to assess their activity, we measured changes in TF activity during differentiation and aging of HSPCs. For that, we took advantage of Single-Cell Regulatory Network Inference and Clustering (SCENIC) approach [38] that calculates the activity of a given TF (regulon score) based on target expression and cis-regulatory elements. We considered 154 TFs, selected from the literature or from our single cell expression data analysis (Seurat cluster markers), out of which, 58 were identified as active regulons in our HSPCs (Additional file 10: Table S9A). By looking at regulon activities of young HSPCs along the trajectory, we revealed a specific regulon signature for each state (Additional file 10: Table S9B). State 1 was characterized with activity of the stress sensors Atf3, the interferon signaling factors, Irf1, Irf7, Irf9, and the downstream targets of the Tgfbeta signaling, Stat1, Klf4, Egr1, Klf6, Junb, depicting a stemness state (regulon clusters C1a and C1b Fig. 5a and C1a Fig. 5b, young panel). Comparison of TF activities between state 2 and state 3 at the first bifurcation (p) emphasized the L fate of state 2 with the detection of high activity of the T cell transcription factors Ikzf1, Sox4 (regulon cluster C2 Fig. 5a, young panel) while state 3 cells enter a more general differentiation program with a slight increase of regulon activities such as Myc (regulon cluster C3 Fig. 5a, young panel). As expected, aging reduced the activity of the two regulons in state 2 witnessing the reduced lymphoid activity during aging. By contrast, Klf6, Junb, Jun, and Stat1 activities of aged HSCs were spread and increased in aged states 1 and 3, (Fig. 5a, b and Additional file 10: Table S9C), which was consistent with the stem cell activity of aged states 1 and 3 containing mainly LTHSCs (Fig. 4b).

By looking at the second bifurcation (pMye) between state 4 and state 5, we confirmed that state 4 was neutrophil- and mast-biased as it was indorsed with a high activity of C/ebpa-e, Runx1, and Irf8, involved in myeloid differentiation (regulon cluster C4 Fig. 5b, young panel). Noticeably, aging decreased the activity of regulons involved in myeloid fate such as Cebpa and -e in state 4 (Fig. 5b and Additional file 10: Table S9C). This result was consistent with the decrease of neutrophils and mastocyte primed-cell number with aging (observed in Fig. 2b) and strengthened our hypothesis that myeloid bias of aged hematopoiesis would not come from this path of the trajectory. Cluster C5 of the heatmap shows that State 5 was characterized with a strong activity of Klf1, E2f8, Ybx1, Gfi1b, and Ezh2, all of which are implicated in the erythroid/megakaryocyte development (regulon cluster C5 Fig. 5b, young panel). Interestingly, the activity of E2f8 was significantly reduced with aging in state 5 whereas Gfi1b activity was considerably increased in this aged state. It should be noted that Gfi1b is the regulon that experienced the greatest increase in activity with aging, not only in state 5, but also at the beginning of the trajectory in state 1. As Gfi1b is a master regulator of thrombopoiesis (reviewed in [39]) and as we found some of its targets such as *Clu*, *Esam*, and *Serpinb1a*, annotated for hemostasis (Additional file 10: Table S9A) upregulated with aging (Additional file 8: Table S7B), we suggested that Gfi1b sustains the platelet bias of aged HSPCs.

Thus, TF activity analyses over the pseudotime corroborated the trajectory features and clearly identified a separation in TF activity that explains the L priming (Fig. 5a) and the two distinct myeloid fates, NeuMast and MkEr (Fig. 5b). It also indicated that aging is associated with marked changes in TF expression and activity with a gain of TFs involved in stemness and platelet activity and a loss of lineage-specific factors that drive lineage commitment and terminal differentiation.

### Cell cycle analysis along pseudotime highlights a delay in differentiation associated with cell cycle arrest in aged condition

**Fig. 6:**
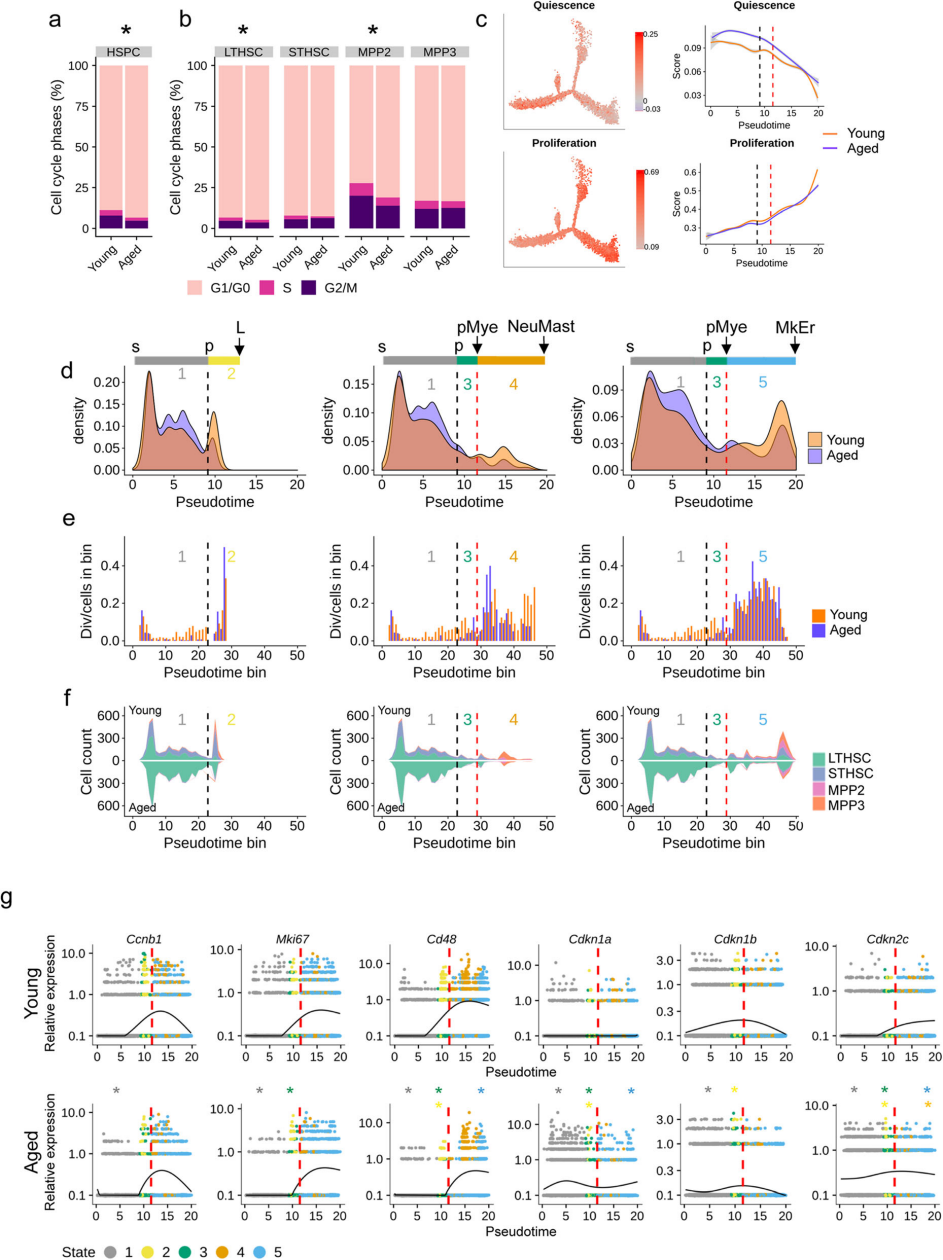
Cell cycle analysis along pseudotime highlights a delay in differentiation associated with cell cycle arrest in aged condition. **a** Repartition (in percentage) of the cell cycle phases (estimated with cyclone) in young and aged HSPCs. **b** Repartition (in percentage) of the cell cycle phases (estimated with cyclone) in LTHSCs, STHSCs, MPP2 and MPP3 in young and aged conditions. For **a** and **b**, * indicates a significant dependence between cell cycle phase and age repartitions (*p* value < 0.05 Pearson’s chi-squared test). **c** Left panel, differentiation trajectory of HSPCs colored in accordance to their score for previously published quiescence and proliferation signatures. Right panel, comparison of the scores for the quiescence and proliferation signatures between young and aged HSPCs in pseudotime. **d** Density plot of young (orange) and aged (purple) cells along pseudotime for the T (left), NeuMast (middle), and MkEr (right) fates. Black and red dashed lines mark respectively p and pMye bifurcation points. **e** Division rate along pseudotime for young (orange) and aged (purple) HSPCs for the T (left), NeuMast (middle), and MkEr (right) fates. On *x*-axis, pseudotime was cut into 50 bins and a division rate is calculated for each bin, by dividing the number of young (*resp*. aged) cells assigned to G2M phase by the total number of young (*resp*. aged) cells of the bin. Black and red stretched lines mark p and pMye pseudotime bifurcation point respectively. **f** Stacked plot of predicted cell types along pseudotime cut into 50 bins for young (upper part of the plots) and aged (lower part of the plots), for the L (left), NeuMast (middle) and MkEr (right) fates. Black and red stretched lines mark p and pMye bifurcation point pseudotime respectively. **g** Smoothed gene expression along pseudotime of selected markers for young (upper panel) and aged (lower panel) HSPCs. Points represent cells, which are colored according to their belonging to the 5 different states (1 gray, 2 yellow, 3 green, 4 orange and 5 blue). The *y*-axis is in log scale. * indicates significant differences in gene expression between young and aged cells (*p* value < 0.05) and star color indicates the state where the difference is found

As one of the hallmarks of HSC aging is a reduction of cycling HSCs [40], we analyzed the cell cycle phases according to BM age. We showed an increase of non-cycling HSPCs (G1/G0) at the expense of the S and G2/M phases in aged BM in comparison to young one (Fig. 6a). Analysis of LTHSC, STHSC, MPP2, and MPP3 population separately showed that age did not typically affect the proportion of cycle phases within each subtype, with the exception of a slight but significant change in LTHSCs and MPP2 (Fig. 6b). This suggests that the increase of the G1/G0 phase proportion observed upon aging is mainly due to the accumulation of quiescent LTHSCs that are known to be arrested in G1/G0 phase [41], and to a lesser extent to LTHSC and MPP2 intrinsic cell cycle changes induced by aging.

Positioning quiescent versus proliferative cells along the trajectories showed that quiescent cells were at the departure of the trajectory while proliferating cells were towards the differentiated states (Fig. 6c, left panel). Comparison of the quiescence and proliferation signatures between young and aged HSPCs showed a quiescence gain in the aged condition in the first part of the trajectory (states 1, 2 and 3) while the proliferation signature remained unchanged (Fig. 6c, right panel and Additional file 11: Table S10A).

Next, we addressed the question of cell cycle and its influence on HSPC aging. We first looked at the distribution of young and aged HSPCs along the trajectory, analyzing T, NeuMast, and MkEr fates separately (Fig. 6d). Doing so, we confirmed the accumulation of aged HSPCs in state 1 before the first bifurcation point p and the decrease of aged cells in the differentiated states 2, 4, and 5 (Fig. 6d). To associate cell-cycle status and cell accumulation, we performed high-resolution analysis of HSC cell cycle along the trajectory by plotting the ratio of dividing cells on pseudotime bins for young and aged cells in Lymphoid, NeuMast, and MkEr fates separately (Fig. 6e). This highlighted a dramatic loss of dividing cells in aged condition in state 1 with the exception of cells located at the very beginning of the trajectory (Fig. 6e). We hypothesized that these dividing cells (that are LTHSCs and belong to np3 cluster) represent cell-cycle activity of self-renewing LTHSCs. Interestingly, we found no difference in cell cycle phase proportion between these young and aged LTHSCs (*p* value > 0.3 Pearson’s chi-squared test; Additional file 1: Fig. S12), suggesting a conservation of self-renewal potential in aged HSCs. By opposition, the absence of cell cycle activity of aged HSPCs later in state 1, which may represent cell cycle activity linked to differentiation, underlines a default in cell division of aged HSPCs associated to differentiation (Fig. 6e). Division rate of aged HSPCs became positive after the first bifurcation and was similar to what we observed in young HSPCs (Fig. 6e), with the exception of a decrease in aged cycling cells in state 4 (towards NeuMast fate) suggesting a default of cell cycle in aged Neu-primed HSPCs. Visualization of the distribution of the different HSPC subsets confirmed the accumulation of aged LTHSCs at the expense of the STHSCs and revealed a dramatic loss of NeuMast-primed cells upon aging (Fig. 6f).

We extracted from our DEG analyses with aging (Additional file 11: Table S10B) DEGs involved in proliferation, differentiation, and cell cycle and analyzed their expression profile in young and aged cells along the trajectory. We observed a pronounced increase in expression of the two proliferation-division genes, *Ccnb1* and *Mki67*, in young HSPCs that was occurring in state 1 concomitant to the increase of the marker of differentiation *Cd48* (Fig. 6g). In aged cells, increase in the expression of these three genes was also detected but was delayed until the branching point pMye suggesting a delay in the commitment of aged HSPCs. To grasp molecular mechanism(s) that could be involved in this delay, we compared cell cycle inhibitor expression across young and aged HSPC trajectories. *Cdkn1a* and *Cdkn2c* were upregulated along the aged trajectory (except in state 4 for *Cdkn2c*) especially in the first part of the trajectory (states 1, 2, and 3) in comparison to young one. By contrast, *Cdkn1b* was downregulated in states 1 and 2 of the aged trajectory (Fig. 6g and Supplemental Table S10B). The change in expression with aging of the three cell cycle inhibitors known to control HSC fate indicates deregulation of cell cycle phases in aged HSCs. It is interesting to note that *Cdkn1a* was found to be a target of Stat1, Jun, and Junb which are themselves targets of the Klf6 regulon (Additional file 10: Table S9A), four regulons whose activities increased with aging in the same range of pseudotime as changes in the level of *Cdkn1a* expression (Figs. 6g and 5b).

Together, these results suggest that aged HSCs have a default in cell cycle, concomitant to a delay in their differentiation program, which occurs before the lineage priming of the HSPCs.

## Discussion

In this study, we questioned the effect of aging on the heterogeneity of HSCs and their properties using scRNA-seq, which provides a powerful method for defining cell subtypes as well as a detailed description of the functional properties specific to these subtypes [42].

At first, the large number of cells analyzed provided us new insights of HSPC heterogeneity, through the identification of 15 distinct HSPC clusters that we divided in two categories, the non-primed clusters by opposition to the lineage-primed clusters composed of low-abundant HSPCs with restricted lineage potential. We identified distinct lineage-primed HSPCs such as HSPCs with mastocytes, neutrophils, erythrocytes, and lymphoid-restricted lineage signatures in addition to the previously reported Mk-restricted HSPCs [8, 11, 27]. The lineage potentials of HSPCs detected in this study, which favors an early HSPC uni-lineage segregation [11, 43], may be the result of our cell cycle correction that diminish cell cycle gene expression noise, a dominant source of transcriptional heterogeneity in the HSC compartment [44]. Our pseudotemporal reconstruction of differentiation trajectories together with our clustering and transcriptional activity analyses highlighted a clear separation in the fates of specific lineage-primed HSPCs and clearly characterized two bifurcation points, revealing three distinct HSPC fates towards Lymphocyte, Neu/Mast, or Mk/Er lineages. In addition, our analysis showed that lineage priming of HSPCs is not delineate by a specific HSPC subset such as LTHSC, STHSC, MPP2, and MPP3 in any instance. Although we showed that Neu priming is clearly stemming from MPP3 and Er priming from MPP2, lineage priming could also arise from a combination of HSPC subsets. This is the case for Mk priming which stems from LTHSC and MPP2 subsets, in line with previous studies [11, 23]. It is also the case for B and T lymphoid potential found in the four subsets of HSPCs, suggesting a lymphoid-priming occurring earlier in the BM and not restricted to the more engaged Flt3-positive MPP4 as previously reported [45]. The fact that we detected lineage primed cells in the very early subset of HSPCs goes in line with a previous studies showing the existence of four distinct and closely related stages of self-renewing LTHSCs in adult BM that stably adopt lineage-restricted fates (platelet, B and T lymphoid, erythroid, and myeloid lineages) despite remaining multipotent [46]. In addition, we were able to distinguish subtle differences in the LT-HSC compartment. Our study delineates a discrete hierarchy of differentiation within the LTHSCs, thanks to the pseudotime and cluster characterization, which posits the np2 cluster as being the most immature one.

If the accumulation of very immature HSCs in the BM of aged individuals is now an accepted criterion of hematopoietic aging, we still do not fully understand what are the characteristics of these aged HSCs and what causes them to accumulate. By looking at the transcriptomic changes at the single cell scale, we confirmed the global increase of the LTHSC fraction within the HSPCs. However, by analyzing our aged HSPCs by clusters or individually, we could demonstrate that HSPCs are not affected uniformly by aging and grasp some interesting aging feature. At first, we showed that the proportion of aged HSPCs in pMast, pNeu pEr, and pL1-primed clusters was decreased while increased in ifn, tgf, np1, and np2 clusters. In addition, young and aged cells were found in the expected ratio in the pMk cluster. This clearly indicates that the platelet and myeloid bias observed upon aging [23] is not due to an amplification of the pool of lineage-primed HSPCs but stem from other HSPC subsets. Secondly, we highlighted some specific amplification of LTHSCs such as LTHSCs with miss-regulated interferon signaling (ifn cluster). As the increase in interferon response with aging in a number of different tissues has been observed [47] and is consistent with the concept of inflammaging [48], this amplification could afford for the myeloid bias observed in aging. Another interesting HSC group that we detected amplified during aging was the cluster of LTHSCs presenting a Tgf signature that may correspond to the accumulation of the HSC subtypes with differential responses to Tgf that was previously identified [49]. These two types of aged HSCs need to be further analyzed but considering their characteristics, it is tempting to hypothesize that their proportion was increased under stress selection pressures to compensate for the loss of mature cell production that occurs upon aging. Moreover, cluster amplification during aging has not been observed in the same way in our different animals, this is particularly true for the amplification of HSCs marked for Tgf, driven by a batch of aged mice. This heterogeneity of aging might witness the emergence of competitive clones that amplify during aging and fit quite well with the clonal hematopoiesis model. In another perspective, the apparition of the pL2-primed cluster that we observed quasi exclusively in the aged BM might also represent clonal evolution. Since this cluster was characterized with the expression of *Trp53inp1*, a gene limiting B-lymphoid differentiation upon aging, it could correspond to an accumulation of aged HSPCs altered in their lymphoid differentiation [28] but resulting for a pressure of immune deficiency.

Pseudotime trajectory analysis led us to address the question concerning the differentiation state of aged LTHSCs, which were thought to accumulate in a more undifferentiated state compared to young LTHSCs [24]. First, the outcome of our analyses is in favor of no difference in term of differentiation state between young and aged LTHSCs as when plotted together along the trajectory the most immature aged cells were not positioned at an anterior pseudotime compared to the young ones. Second, our results support that aged LTHSCs are delayed in their differentiation journey in comparison to young ones and that this delay occurs pretty early in the pseudotime, before the first bifurcation point that splits lymphoid fate from myeloid fate. This was clearly emphasized by our regulon activity analysis of transcription factors such as Myc, Trp53, or Spi1 that were previously described involved in multipotency and commitment of HSCs [50] and for which we could observe a delay in their activity along the differentiation trajectory.

Thus, the aged HSCs are not more undifferentiated than the young ones but seem to have intrinsic defaults that would delay their commitment. This finding is interesting when putting in perspective what causes the accumulation of LTHSCs. Increase of LTHSCs with aging could originate from an increase in the self-renewal rate of HSCs or/and from a blockade or at least a slowdown of the LTHSCs along their differentiation journey. It was also hypothesized that label-retaining HSCs (LR-HSCs), which divide minimally over time, accumulate in aged BM after completing four traceable symmetric self-renewal divisions to expand its size before entering a state of dormancy [51]. Although we could not directly address the question of self-renewal, we can argue based on our regulon and cell cycle analyses that aged LTHSCs have kept their capacity to self-renew and have not reached a state of complete dormancy but have reduced their proliferation linked to differentiation. Interestingly, we could associate this reduced and age-related proliferation/differentiation potential to a high level of Mycn activity, known to contribute to the stemness and self-renewal of different stem cells [52] and a high level of Gfi1b activity known to promote self-renewal of HSCs [53].

One interesting outcome of our analysis is the link between the delay in differentiation and cell cycle activity changes of aged HSPCs. We deduced from our computational cell cycle classification that lineage-primed HSPCs were less in G1/G0 than the non-primed LTHSCs. This observation is fully consistent with current knowledge that the most undifferentiated HSCs reside in the G0 phase and cycle infrequently and that cell cycle overall becomes more frequent as HSCs are gradually committed [41, 54]. In addition, we detected an increase in HSCs in G1/G0 phases in older BMs and an increase in older LTHSCs in G1/G0 phases compared to younger LTHSCs, which reflects the decrease in cell cycle activity of older HSCs when considered as a whole [55]. Finally, when calculating a division rate per cells and studying division gene expression along the trajectory, we could detect a loss of aged dividing HSPCs located before the first bifurcation of the differentiation trajectory. These cells partially overlap in our trajectory with the div cluster, marked by genes related to asymmetric division such as *gpsm2*, *Ragcap*, and *Ccnb1* [56, 57], suggesting that the delay in differentiation could be linked to an altered capacity of aged HSPCs to divide asymmetrically. In addition, gene expression of cell cycle inhibitors clearly shows that HSPCs in the first part of the trajectory have increased expression in *Cdkn1a* and *Cdkn2c*, promoters of quiescence but a reduction in *Cdkn1b* activation, which promotes commitment [58]. Interestingly, our analysis pointed out *Cdkn1a* as a direct target of Junb, itself target of Klf6. As the activation of *Cdkn1a* by Junb has been previously described to limit hematopoietic stem cell proliferation [59] and as Klf6 is a key factor in Tgfbeta signaling pathway [60, 61], our work unveils an interesting pathway controlled by the cytokine Tgfbeta involving Klf6 as a key regulon and *Cdkn1a* as a cell cycle regulator that is enhanced upon aging, endorses quiescence, and limits HSC differentiation.

## Conclusions

Our single-cell transcriptome-based identification of cell identity and its modifications associated with aging provides new information on cellular heterogeneity and intrinsic changes that will be useful for future investigation of the role of other regulators on the aged HSC phenotype.

## Methods

### Mouse model and cell sorting

C57BL/6-CD45.2 mice were purchased from Charles River Laboratories and were aged at the CRCM animal facility under specific pathogen-free conditions and handled in accordance with the French Guidelines for animal handling (Agreement #02294.01). Only males were analyzed, at 2–3 months (young) and 17–18 months (aged) of age. HSPCs were collected from the BM of 5 young and 5 aged mice over 2 independent batches with cells from 2 pooled young (Young_A sample) and 3 pooled aged (Old_A sample) mice for one batch, and cells from 3 pooled young (Young_B sample) and 2 pooled aged (Old_B sample) mice for the other one (Supplemental Table S1). For each sample, the BM was lineage depleted by using the Lineage Cell Depletion Kit (Miltenyi Biotec) and labeled with the following antibody cocktail: anti CD45.2, anti Sca-1, anti-cKit, anti CD150, anti Cd48, anti Cd34, and anti Flt3 antibodies (Additional file 12: Table S11) to purify Lin-Sca1+cKit+ Flt3 cells (HSPCs) by multi-parameter fluorescence-activated cell sorting (FACS) on a FACSAriaII (SpecialOrderResearch Products; BD Biosciences). Flow cytometry analyses were performed using a BD-LSRII cytometer and analyzed using BD-DIVA Version 6.1.2 software (Special Order Research Products; BD Biosciences).

### Single cell RNA-seq and data processing

We used the 10x genomics platform from two facilities: HalioDX for samples Young_A and Old_A (Marseille, France) and TGML for samples Young_B and Old_B (Marseille, France). In both facilities, FACS purified HSPCs were loaded 30 min after the sorting onto a Chromium Single Cell Chip and processed with the Chromium Controller (10x Genomics) according to the manufacturer’s instructions for single cell barcoding at a target capture rate of 4000 individual cells per sample. Libraries were prepared using Chromium Single-Cell 3′ Reagent Kits v2 (10x Genomics) and were sequenced using an Illumina NextSeq500 sequencer to an average depth of about 45,000 reads per cell for Young_A and Old_B samples and about 130,000 reads per cell for Old_A and Young_B samples. Cell ranger software v2.2 was used to align reads to the (GRCm38) mm10 mouse reference genome. Cell counts and transcript detection rates are summarized in Supplemental Table S1.

### Quality control and data normalization

Cells outside 2 standard deviations (SDs) from the mean UMI log-counts were filtered out for each sample to discard poor quality cells and doublets. In total, 7433 young and 7482 aged cells were kept. For each dataset (our four samples and the Rodriguez-Fraticelli dataset), genes with no UMI count in more than 0.5% of the cells were discarded. All gathering, 17,513 genes were kept. Then, UMI counts were normalized with the NormalizeData Seurat function. For each cell, we considered the log transformation of the ratio of UMI counts per gene by the total UMI counts of the cell, multiply by a scaling factor of 10,000 (log (10,000(UMI_gene_/UMI_cell_) + 1)).

### Cell cycle phase classification

Prediction of cell cycle phase for each cell was made with the cyclone [62], which relies on a pre-defined classifier for cell division constructed from a training dataset of synchronized mouse embryonic stem cells [63]. For each cell, a score based on raw count data before gene filtering was computed for each phase (G2/M, S and G1) and used to assign a phase to the cells. As quiescent HSCs are closer transcriptionally to G1 than S or G2/M cells of the cyclone training dataset, we classified them with the cyclone G1 cells and named this category G1/G0.

### HSPC subtype assignment

In order to assign known FACS cell identity in our HSPC pool, we used CaSTLe (Classification of single cells by transfer learning), a supervised classification method consisting in labeling cells in a scRNA-seq experiment, using knowledge learnt from other experiments on similar subtypes [64]. We chose as source dataset a published scRNA-seq dataset obtained from FACS isolated HSPCs [11]. Cells from this data set (approximately 2000/per type) were divided into 4 subsets: the LTHSC (Lin- Sca1^+^ Kit^+^ Flt3^−^ Cd150^+^ Cd48^−^), the STHSC (Lin- Sca1^+^ Kit^+^ Flt3^−^ Cd150^−^ Cd48^−^), the MPP2s (Lin- Sca1^+^ Kit^+^ Flt3^−^ Cd150^+^ Cd48^+^), and the MPP3 (Lin- Sca1^+^ Kit^+^ Flt3^−^ Cd150^−^ Cd48^+^). HscScores were computed as previously described [35].

### Dataset integration, data scaling, and cell cycle regression

To minimize batch effect between datasets, we integrated our 4 sample datasets (Young_A, Young_B, Old_A, Old_B) following the procedure of Seurat 3 [25]. Integration was done also for young and aged conditions separately. Briefly, the highly variable genes for each dataset were selected with the FindVariableFeatures function (selection.method =“vst”) and ranked according to the number of datasets in which they were independently identified as highly variable. The top highly variable 2000 genes were thus integrated by iteratively merging pairs of datasets according to a given distance. Integration anchors, representing two cells that are predicted to originate from a common biological state in both datasets using a Canonical Correlation Analysis (CCA), were done using the FindIntegrationAnchors function (dims=1:15). Then, the expression of the target dataset was corrected using the difference in expression between the two expression vectors for each pair of anchor cells. This step was realized using IntegrateData function (dims=1:15). This process resulted in an expression matrix that contains the batch-effect-corrected expression for the 2000 selected genes of the cells from the 4 samples.

Standardized (i.e., centered and reduced) expression values with cell to cell variations due to cell cycle effect regressed were obtained with the ScaleData function of Seurat using the G2/M, S and G1/G0 scores previously computed for each cell by cyclone for the var.to.regress argument (cf Cell cycle phase classification**)**.

### Dimension reduction and clustering

A PCA was performed on the scaled data using RunPCA Seurat function (npc = 40). The 15 first principal components of the PCA were kept for nonlinear dimension reduction and cell clustering. Uniform Manifold Approximation and Projection (UMAP), [26], a nonlinear dimension reduction method, was run using RunUMAP Seurat function package in order to embed cells in a 2-dimensional space. A K-nearest neighbor graph (KNN) based on the Euclidean distance in PCA space was constructed (*k* = 20) to cluster the cells with the Louvain algorithm (resolution = 0.5) using the FindNeighbors and FindClusters Seurat functions respectively.

### Pseudotime ordering

Unsupervised ordering of the HSPCs was done with the Seurat 3 integrated results as input to build a tree like differentiation trajectory using the DDRTree algorithm of the R package Monocle v2 [31]. Integrated data from (i) all samples (young and aged) excluding the primed pL2 cluster cells, (ii) young cells only, or (iii) aged cells only were processed with Monocle. For the three pseudotime ordering analyses (all cells, young only, and aged only), the 2000 gene expression matrix, scaled and regressed for cell cycle effect (see *Data scaling and cell cycle regression*) issued from the Seurat 3 integrated samples was loaded into Monocle using the newCellDataSet function (lowerDetectionLimit = 0.1, expressionFamily = uninormal()). The 2000 genes were set as ordering genes and trajectory building was made by calling the reduceDimension Monocle function (max_components = 2, reduction_method = ‘DDRTree’, norm_method = “none”, pseudo_expr =0). For each of the three trajectories, the root state was identified by selecting the Monocle state with the highest proportion of LTHSC predicted subtype (Fig. 4b; Additional file 1: Fig. S6B) in order to compute pseudotime values for the cells using the orderCells Monocle function. Expression of some genes as a function of pseudotime (Fig. 6g) was plotted with the plot_gene_expression Monocle function (using the Monocle normalization method with the estimateSizeFactor Monocle function).

### Differential gene expression analyses

Specific markers for each cluster (Additional file 3: Table S2) and for each Monocle state (Additional file 9: Table S8B & C) were identified using FindAllMarkers Seurat function, with default parameters on log-normalized data without any cell cycle correction. Genes significantly overexpressed in one cluster/state versus all the others (positive markers) were tested with Wilcoxon rank sum tests on the log-normalized data of the given cluster against all the others. To further characterize state 2, which shared 72% of its markers with state 4, we identified DEGs between the two states using FindMarkers Seurat function (Additional file 9: Table S8C). Only genes expressed in at least 10% of the cells in either of the two groups (min.pct = 0.1) and with a log fold change threshold of 0.25 (logfc.threshold = 0.25) were tested. A *p*-adjusted value (Bonferroni correction) threshold of 0.05 was applied to filter out non-significant markers.

Aging markers for the global population were obtained with the FindConservedMarkers Seurat function (min.pct = 0.1, logfc.threshold = 0) using the sequencing platform as grouping variable to minimize batch effect (Young_A, Old_A were processed on HalioDx platform and Young_B, Old_B on TGML platform). The Wilcoxon rank-sum test was performed on the log-normalized data between all young versus all aged cells (Additional file 6: Table S5) from each batch separately and the two *p* values for each gene were combined using the Tipett’s method. Genes presenting an opposite variation between the 2 batches were filtered out.

Aging markers for each cluster (Additional file 7: Table S6) and for each Monocle state (Additional file 11: Table S10B) were obtained with the same method by looking at the difference cluster per cluster and state per state (min.pct = 0.1, logfc.threshold = 0.25 for each cluster and min.pct = 0, logfc.threshold = 0 for each state). No tests were performed in the pL2 cluster cells because it contained less than 3 cells in one young pool. From these results, for each cluster and each state only significant aging markers (combined *p* value < 0.05 and same direction of variation in the 2 batches) were kept.

Among these markers the highly variable ones (average log fold change > 0.5 with aging in at least one cluster in both batches) were selected to generate heatmap for all clusters with primed clusters gathered (Fig. 3a) and for primed clusters only (Additional file 1: Fig. S4) by adapting the DoHeatmap Seurat function. Genes (raw) were ordered using hclust R function on standardized aging gene expression of the subset. Euclidian distance and unweighted pair group method with arithmetic mean (UPGMA) were used. Up- and downregulated genes with aging were ordered separately.

Volcano plots for the global aging markers were drawn (Additional file 1: Fig. S3) with EnhancedVolcano function from the R package of the same name [65].

### Gene set enrichment analysis

To characterize the identified clusters with Seurat, we performed gene set enrichment analysis on cluster markers with g:Profiler v0.6.7 [66] with default arguments except for background set to all genes expressed in the whole dataset (i.e., genes that passed filtering during quality control). We tested enrichments in GO terms (GO:BP, GO:MF, GO:CC) as well as in terms from KEGG, REAC, TF, MI, CORUM, HP, HPA, and OMIM databases (Fig. 1c and Additional file 4: Table S3). Cluster markers were also tested for enrichment in previously published gene set signatures related to HSPCs (Additional file 13: Table S12). Signatures tested were Bcell_Chambers, Diff_Chambers, Gran_Chambers, HSC_Chambers, Lymph_Chambers, Mono_Chambers, Mye_Chambers, NK_Chambers, NaiveT_Chambers, and Ner_Chambers [32], lineage priming of HSC signatures C1, C2, C3, Mk, Er, Ba, Neu, Mo, Mo2, preDC, preB and preT [11], and HSCs and aging signatures Mm_HSC_Runx1_Wu, Mm_HSC_Tcf7_Wu [67], Mm_LT_HSC_Venezia, Mm_Proliferation_Venezia, Mm_Quiescence_Venezia [68], Polarity_factors_Ting, Novel_HSC_regul_polar_Ting [57], HSC aging Svendsen [30] and MGA-MEP [34]. Cluster marker enrichment for the different signatures in comparison to all dataset genes was tested using a hypergeometric test (phyper R function). To perform enrichment analysis of aging markers with a consistent gene number, we gathered the overexpressed (resp. underexpressed) markers from at least one cluster and used gprofiler as describe above (Additional file 8: Table S7A & B). Expression scores of the signatures or of selected aging features from the enrichment analysis were calculated for each individual cell using the AddModuleScore Seurat function (on log-normalized data) with default parameters, using as input the genes of the signatures or the aging markers annotated for the selected features. The Svendsen signature score was computed in the same way taking the aging markers common to our study and those of Svendsen’s re-analysis [30].

### Differential signature score analysis

Signature markers of Monocle state were tested in the same way as gene state markers (see above) using FindAllMarkers (min.pct = 0, logfc.threshold = 0) with Student’s *t* tests. Only signatures with an average score differences above 0.015 between one state and all were kept. A *p*-adjusted value (Bonferroni correction) threshold of 0.05 was applied to filter out non-significant differences.

Signature score differences with aging in each state were tested in the same way as the aging markers per clusters (see above) using the FindConservedMarkers Seurat function (sequencing platform as grouping variable, min.pct and logfc.threshold set to 0) with Student’s *t* tests. For each Monocle state, only average score differences of same sign and above 0.015 in the two batches presenting a combined *p* value < 0.05 were kept (Additional file 9: Table S8A).

The selected aging features expression score differences with aging in each cluster were tested in the same way as the aging markers per clusters (see above) using the FindConservedMarkers Seurat function (sequencing platform as grouping variable, min.pct and logfc.threshold set to 0) with Student’s *t* tests (Additional file 8: Table S7C). For each cluster, only average score differences of same sign and above 0.1 in the two batches presenting a combined p value < 0.05 are considered as significant (Fig. 3b). No tests were performed in the primed B cells clusters because it contained less than 3 cells in one young pool.

### Regulon analysis

pySCENIC (1.10.0) was used with its command line implementation [38]. The raw expression matrix for the cells of all samples was filtered, by keeping genes with a total expression greater than 2*0.01*(number of cell). 10,698 genes passed the filtering. pyscenic grn command was used with grnboos2 method and default options and a fixed seed to derive co-expression modules between transcription factors and potential targets. We used as input all the markers of the Seurat clusters for which a transcription factor binding motif was available in the motifs-v9-nr.mgi-m0.001-o0.0 database provided by Scenic, plus several TFs involved in early hematopoiesis, Spi1, Tal1, Zfpm1, Cbfa2t3, Erg, Fli1, Gata1, Gata2, Hhex, Runx1, Smad6 [69], Gfi1b [70], and Zbtb16 [71]. The obtained modules were refined by pruning targets that did not have an enrichment for a corresponding motif of the TF with pyscenic ctx command with –maskdropouts option using the motif database motifs-v9-nr.mgi-m0.001-o0.0 and the cis-target database mm9-tss-centered-10 kb-7species.mc9nr. Only positive regulons (i.e., those with a positive correlation between the TF and its targets) were kept for downstream analysis (Additional file 10: Table S9A). AUCell scores (regulon activities) in each cell were computed with pycenic aucell command (default options). To be noted that number of target genes was highly variable from a regulon to another (Additional file 1: Fig. S9).

For young and aged HSPCs, two heatmaps of regulon activity scores, along pseudotime, were made in order to analyze transcriptional activity at the two bifurcation points for both ages. See Additional file 10; Supplementary methods for detailed regulons heatmaps construction.

### Analysis of HSPC subtypes and cell cycle phases in the differentiation trajectory depending on age

Cell density (Fig. 6d), division rate (Fig. 6e), and stacked plot of HSPC subtypes (Fig. 6f) were computed and plotted along pseudotime at each age for the 3 HSPC fates: the lymphoid (Monocle states 1 and 2), the Mastocytes/Neutrophils (Monocle states 1, 3, and 4), and the Megakaryocytes/Erythrocytes (Monocle states 1, 3, and 5). For division rate and stacked plot of HSPC subtypes, pseudotime was cut into 50 bins. For each age, in each pseudotime bin, division rate was computed as the ratio of the number of cells with a G2/M phase assigned to the total number of cells in the bin.

### Statistics

Statistics were computed with R software v3.5.1. The statistical tests for gene expression and signature or regulon activity scores were performed with Seurat and are detailed above. In each cluster and in non-primed/primed clusters gathered, the enrichment of age was tested using a hypergeometric test (phyper R function Fig. 2b). Chi^2^ tests (chisq.test R function) were performed to test independence between cell cycle phase and age, in all cells (Fig. 6a) and in each HSPC subtype separately (Fig. 6b), and in the cells at the departure of the trajectory (Pseudotime < 2, Additional file 11: Fig. S10) and to test independence between Monocle state and age in all Monocle states (Fig. 4i) and in the states 2, 3, and 5 only (Fig. 4j). Fisher’s exact test (fisher.test R function) was performed to test independence between Monocle state and age in each Seurat cluster (Additional file 9: Supplemental Fig. S8B). Wilcoxon rank-sum test was used to test for median difference between pseudotime value distributions of young and aged cells (Fig. 4h). In each cluster, a linear regression was computed between the average log fold change (in the cluster) and the global (in all cells) average log fold change of the aging markers recovered in the cluster (lm R function Additional file 6: Supplemental Fig. S5). Smooth curves of module score expression in pseudotime through the different fates for young and aged cells were drawn for quiescence and proliferation signature (Fig. 6c) using the geom_smooth function ggplot2 R package [72] with the gam function of mgcv R package [73].

## Supplementary Information

**Additional file 1.** **Supplementary Methods.**

**Additional file 2: Table S1.**

**Additional file 3: Table S2.**

**Additional file 4: Table S3.**

**Additional file 5: Table S4.**

**Additional file 6: Table S5.**

**Additional file 7: Table S6.**

**Additional file 8: Table S7.**

**Additional file 9: Table S8.**

**Additional file 10: Table S9.**

**Additional file 11: Table S10.**

**Additional file 12: Table S11.**

**Additional file 13: Table S12.**

## Data Availability

The single-cell RNA-seq data generated here are available in the Gene Expression Omnibus database under accession code GSE147729 [74]**.** All R and python codes used for data analysis are integrated in a global snakemake workflow available at: https://gitcrcm.marseille.inserm.fr/herault/scHSC_herault [75].
