# Author Correction to: Single-cell RNA-seq reveals a concomitant delay in differentiation and cell cycle of aged hematopoietic stem cells

**DOI:** 10.1186/s12915-021-01005-4

**Published:** 2021-04-16

**Authors:** Léonard Hérault, Mathilde Poplineau, Adrien Mazuel, Nadine Platet, Élisabeth Remy, Estelle Duprez

**Affiliations:** 1 Epigenetic Factors in Normal and Malignant Hematopoiesis Team, Aix Marseille Université, CNRS, INSERM, Institut Paoli-Calmettes, CRCM, Marseille, France; 2 Aix Marseille Université, CNRS, Centrale Marseille, I2M, Marseille, France

**Correction to: BMC Biology 19, 19 (2021)**

**https://doi.org/10.1186/s12915-021-00955-z**

Following publication of the original article [1], the authors noted that Additional file 1 was missing the supplemental Figures S1-S12. The corrected version of Additional file 1 is attached to this Author Correction and the Additional file 1 has been updated in the original article accordingly.

## Supplementary Information

**Additional file 1: Supplementary Methods. Figure S1.** LTHSCs accumulate upon aging. (A) FACS profiles of young and aged HSPCs. (B-D) Cell type classification: Proportions of LTHSC, STHSC, MPP2 and MPP3 determined by FACS and by supervised classification with CaSTLe when considering (B) all HSPCs, (C) young and aged HSPCs separately and (D) the 4 samples separately. **Figure S2.** Representative gene markers used to identify HSPC clusters. Violin plots showing gene markers expressed by the 15 clusters revealed in the UMAP shown in Fig. 1b. The complete list of significantly up-and down-regulated genes for the 15 clusters is shown in Supplemental Table S2. **Figure S3.** Violin plots showing Ly6d and Trp53inp1 expression significantly up regulated in the pL2 cells cluster in comparison to the other cells (*p*-value < 0.05 & log fold change > 0.25). **Figure S4.** Volcano plot of differential expression upon aging tested on all cells. Black dots indicate significant DEGs (*p*-value < 0.05 and log fold change > 0.25). A total of 3362 genes were tested. **Figure S5.** Heatmap of the most significant differentially expressed genes upon aging (p-value < 0.05 and log fold change > 0.5 in at least one cluster) in the 6 lineageprimed clusters revealed by the Seurat analysis (Fig. 1b). Gene expression is standardised across the entire dataset. **Figure S6.** Comparison of cluster gene expression changes with global gene expression changes upon aging. For each significant aging marker in a given cluster (*p*-value < 0.05 and log fold change > 0.25), its global log fold change (logFC; x-axis) is plotted with its cluster log fold change (logFC; y-axis). For each cluster, a regression line is drown in blue, formula is indicated at the left top corner with its square regression coefficient R2. **Figure S7.** (A) Monocle trajectories for young and aged HSPCs ordered separately. Cells are coloured according to their belonging to the 3 states (6 grey, 7 yellow, 8 blue) or to the pL2 cluster (brown). Both trajectories present a similar segregation between the lineage-primed HSPCs, with one bifurcation from LTHSC (state 6) towards Neu/Mast-primed (NeuMast) HSPCs (state 7) and Mk/Erprimed (MkEr) HSPCs (state 8). The bifurcation to the lymphocyte fate was not retrieved probably due to the reduction in pL1 cell number due to sample splitting. (B) Barplots representing the LTHSC, STHSC, MPP2 and MPP3 proportions in the three states. (C) Monocle trajectories of young and aged HSPCs coloured in accordance to their pseudotime values and representing their differentiation progression. (D) Repartition of the Seurat clusters along the pseudotime of young and aged HSPC trajectories. Box plots of pseudotime values are coloured according to the most represented state. (E) Repartition (in percentage) of the different states (6 to 8) of the trajectory for each Seurat cluster for young and aged HSPCs. **Figure S8.** Localization of the different Seurat clusters in Monocle trajectory. Cells belonging to a given cluster are coloured in orange for young and in purple for aged HSPCs. **Figure S9.** Analysis of the hscScore according to Seurat clusters. Violin plots of hscScore distribution is presented in the 15 clusters. **Figure S10.** Repartition of young and old HSPCs in Monocle pseudotime and in states per Seurat cluster. (A) Boxplots of Monocle pseudotime values of the young (dark) and aged (pale) cells from the different clusters obtained with Seurat (except pL2 cluster). Box plots showing medians are coloured according to the most represented state. (B) Comparison of Monocle state percentage in the different clusters between young (Y, dark colours) and aged (A, pale colours). Stars indicate a significant dependence between state repartition of the cells and age (*p*-value < 0.05 Fisher’s Exact Test). **Figure S11.** Number of targets recovered for each regulon identified with scenic**.** Y axis is in log scale. **Figure S12.** Young and aged HSPCs located at the very beginning of the trajectory cycled the same. (A) Highlight in the trajectory of the starting cells (coloured in black, pseudotime < 2). (B) Cell cycle phase prediction of young and aged starting cells highlighted in A. NS: no significant dependence between age and phase repartition (*p*-value > 0.3 Pearson’s Chi-squared test).
