# Supplementary material for: Single-cell RNA-seq reveals a concomitant delay in differentiation and cell cycle of aged hematopoietic stem cells
Source: BMC Biol. 2021 Feb 1;19:19. doi: 10.1186/s12915-021-00955-z (PMC7851934; doi:10.1186/s12915-021-00955-z)
Supplement: Supplementary file 1 — Additional file 1. Supplementary Methods. [file 12915_2021_955_MOESM1_ESM.pdf]

# ADDITIONAL FILE 1

## SUPPLEMENTARY METHODS

**Regulon heatmaps**

The first heatmaps measured the regulon activity from the departure of the trajectory (state 1) towards the ends of Monocle state 2 and Monocle state 3 (Figure 5a). The second ones measured the regulon activity from the departure of the trajectory (excluding state 2) towards Monocle state 4 and Monocle state 5 extremities (Figure 5b). Thus, each heatmap displays one bifurcation point and two paths. First, for each path at each age, a generalized linear model is fitted to the activity scores of each regulon as a function of pseudotime using the gam function of mgcv R package (1). Pseudotime is cut into 100 bins of equal length. For each bin and for each regulon the mean of the regulon activity score on all cells belonging to the bin is computed. The resulted matrix with data from the two paths for the two ages was scaled and regulons were hierarchically clustered on the young data subset thanks to the hclust R function using Euclidian distance and ward.D2 clustering method (4 clusters for the first and the second heatmaps). The regulon order obtained was then used to build the final heatmap on all the data with the pheatmap of pheatmap R package (2). Regulon markers of monocle states were tested in the same way as gene state markers (see above) with their AUCell scores using FindAllMarkers Seurat function (min.pct= 0.1, logfc.threshold=0) with Wilcoxon rank sum tests. Only regulons with an average AUCell score differences above 0.002 between one state versus all the others were kept. A *p*-adjusted value (Bonferroni correction) threshold of 0.05 was applied to filter out non-significant differences.
Regulon activity differences with aging in each state were tested in the same way as the aging markers per clusters using the FindConservedMarkers Seurat function (sequencing platform as grouping variable, min.pct = 0.1 and logfc.threshold = 0) with Wilcoxon rank sum tests. For each state, only average AUCell score differences of same sign and above 0.002 in the two batches presenting a combined p value < 0.05 were kept (Supplementary Table 9B).


(1) Wood, S.N., Pya, N., and Säfken, B. (2016). Smoothing Parameter and Model Selection for General Smooth Models. Journal of the American Statistical Association *111*, 1548-1563.
(2) Kolde (2019). pheatmap: Pretty Heatmaps. R package version 1.0.12. (https://CRAN.R-project.org/package=pheatmap).
