# Supplementary material for: Single-cell RNA-seq reveals a concomitant delay in differentiation and cell cycle of aged hematopoietic stem cells
Source: BMC Biol. 2021 Feb 1;19:19. doi: 10.1186/s12915-021-00955-z (PMC7851934; doi:10.1186/s12915-021-00955-z)
Supplement: Supplementary file 12 — Additional file 12: Table S11. [file 12915_2021_955_MOESM12_ESM.docx]

| **Antibodies** | **Fluorochrome** | **Source/identifier** |
| --- | --- | --- |
| Lineage-SAV | PECF594 | BD pharmingen |
| Sca1 | PerCP-Cy5.5 | Biolegend/E11.1641.7 |
| CD117/cKit | APC-eF780 | eBioscience/288 |
| CD150 | APC | Biolegend/TC15-12F12.2 |
| CD48 | BV421 | eBioscience/HM48-1 |
| CD34 | FITC | eBioscience/Ram34 |
| CD135/Flt3 | PE | Biolegend/AF10 |
| CD45.2/Ly5.2 | AF700 | Biolegend/104 |

**Supplementary Table 11: List of antibodies used for cytometry**
