# Supplementary material for: Author Correction to: Single-cell RNA-seq reveals a concomitant delay in differentiation and cell cycle of aged hematopoietic stem cells
Source: BMC Biol. 2021 Apr 16;19:80. doi: 10.1186/s12915-021-01005-4 (PMC8052674; doi:10.1186/s12915-021-01005-4)
Supplement: Supplementary file 1 — Additional file 1: Supplementary Methods. Figure S1. LTHSCs accumulate upon aging. (A) FACS profiles of young and aged HSPCs. (B-D) Cell type classification: Proportions of LTHSC, STHSC, MPP2 and MPP3 determined by FACS and by supervised classification with CaSTLe when considering (B) all HSPCs, (C) young and aged HSPCs separately and (D) the 4 samples separately. Figure S2. Representative gene markers used to identify HSPC clusters. Violin plots showing gene markers expressed by the 15 clusters revealed in the UMAP shown in Fig. 1b. The complete list of significantly up-and down-regulated genes for the 15 clusters is shown in Supplemental Table S2. Figure S3. Violin plots showing Ly6d and Trp53inp1 expression significantly up regulated in the pL2 cells cluster in comparison to the other cells (p-value < 0.05 & log fold change > 0.25). Figure S4. Volcano plot of differential expression upon aging tested on all cells. Black dots indicate significant DEGs (p-value < 0.05 and log fold change > 0.25). A total of 3362 genes were tested. Figure S5. Heatmap of the most significant differentially expressed genes upon aging (p-value < 0.05 and log fold change > 0.5 in at least one cluster) in the 6 lineageprimed clusters revealed by the Seurat analysis (Fig. 1b). Gene expression is standardised across the entire dataset. Figure S6. Comparison of cluster gene expression changes with global gene expression changes upon aging. For each significant aging marker in a given cluster (p-value < 0.05 and log fold change > 0.25), its global log fold change (logFC; x-axis) is plotted with its cluster log fold change (logFC; y-axis). For each cluster, a regression line is drown in blue, formula is indicated at the left top corner with its square regression coefficient R2. Figure S7. (A) Monocle trajectories for young and aged HSPCs ordered separately. Cells are coloured according to their belonging to the 3 states (6 grey, 7 yellow, 8 blue) or to the pL2 cluster (brown). Both trajectori [file 12915_2021_1005_MOESM1_ESM.pdf]

ADDITIONAL FILE 1

SUPPLEMENTARY METHODS

Regulon heatmaps

The first heatmaps measured the regulon activity from the departure of the trajectory (state 1) towards the ends of Monocle state 2 and Monocle state 3 (Figure 5a). The second ones measured the regulon activity from the departure of the trajectory (excluding state 2) towards Monocle state 4 and Monocle state 5 extremities (Figure 5b). Thus, each heatmap displays one bifurcation point and two paths. First, for each path at each age, a generalized linear model is fitted to the activity scores of each regulon as a function of pseudotime using the gam function of mgcv R package (1). Pseudotime is cut into 100 bins of equal length. For each bin and for each regulon the mean of the regulon activity score on all cells belonging to the bin is computed. The resulted matrix with data from the two paths for the two ages was scaled and regulons were hierarchically clustered on the young data subset thanks to the hclust R function using Euclidian distance and ward.D2 clustering method (4 clusters for the first and the second heatmaps). The regulon order obtained was then used to build the final heatmap on all the data with the pheatmap of pheatmap R package (2). Regulon markers of monocle states were tested in the same way as gene state markers (see above) with their AUCell scores using FindAllMarkers Seurat function (min.pct= 0.1, logfc.threshold=0) with Wilcoxon rank sum tests. Only regulons with an average AUCell score differences above 0.002 between one state versus all the others were kept. A *p*-adjusted value (Bonferroni correction) threshold of 0.05 was applied to filter out non-significant differences.
Regulon activity differences with aging in each state were tested in the same way as the aging markers per clusters using the FindConservedMarkers Seurat function (sequencing platform as grouping variable, min.pct = 0.1 and logfc.threshold = 0) with Wilcoxon rank sum tests. For each state, only average AUCell score differences of same sign and above 0.002 in the two batches presenting a combined p value < 0.05 were kept (Supplementary Table 9B).

(1) Wood, S.N., Pya, N., and Säfken, B. (2016). Smoothing Parameter and Model Selection for General Smooth Models. Journal of the American Statistical Association *111*, 1548-1563.
(2) Kolde (2019). pheatmap: Pretty Heatmaps. R package version 1.0.12. (https://CRAN.R-project.org/package=pheatmap).

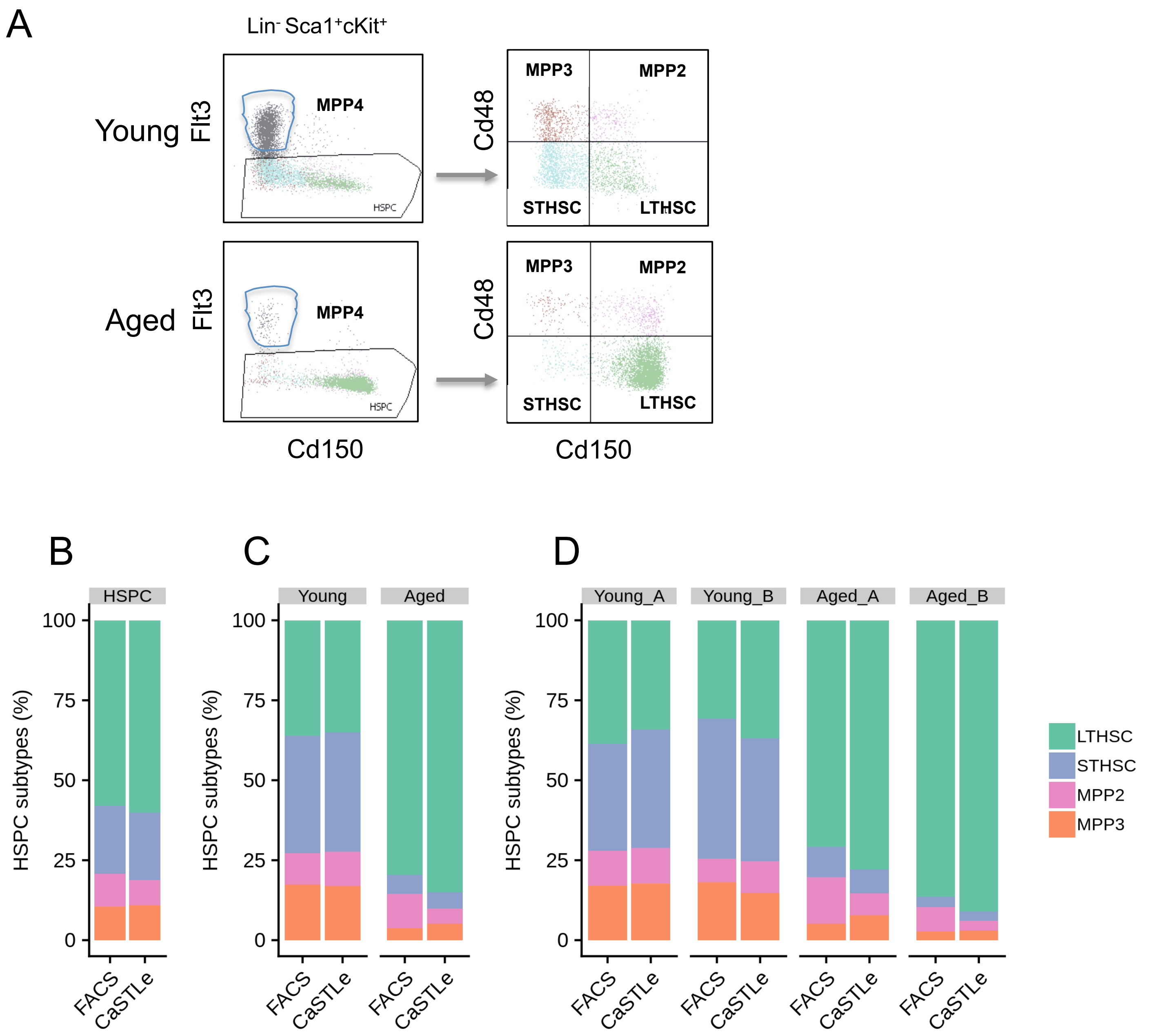

Supplemental Figure S1: LTHSCs accumulate upon aging. (A) FACS profiles of young and aged HSPCs. (B-D) Cell type classification: Proportions of LTHSC, STHSC, MPP2 and MPP3 determined by FACS and by supervised classification with CaSTLe when considering (B) all HSPCs, (C) young and aged HSPCs separately and (D) the 4 samples separately.

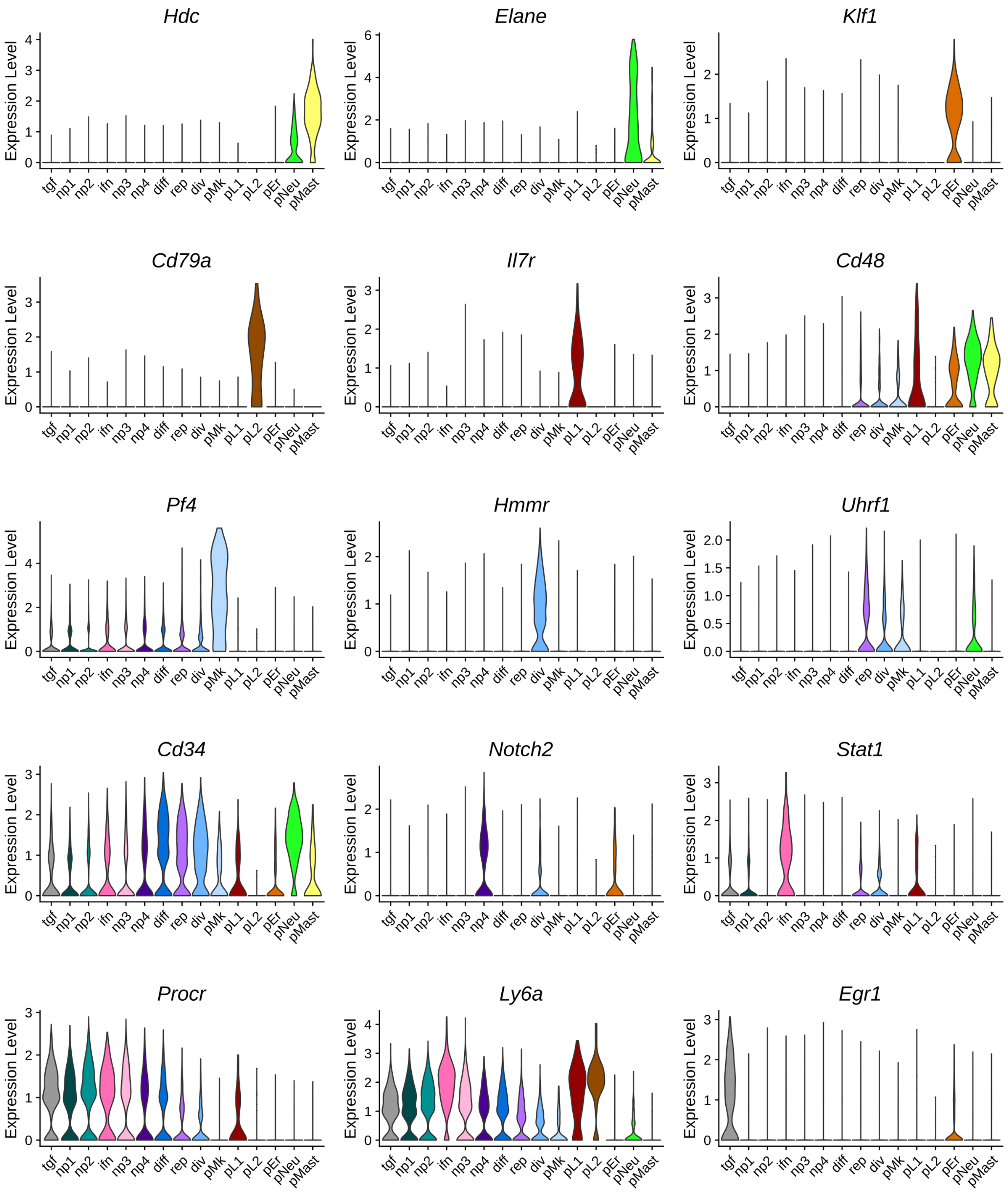

Supplemental Figure S2. Representative marker genes used to identify HSPC clusters. Violin plots showing gene markers expressed by the 15 clusters revealed in the UMAP shown in Fig. 1b. The complete list of significantly up-and down-regulated genes for the 15 clusters is shown in Supplemental Table S2.

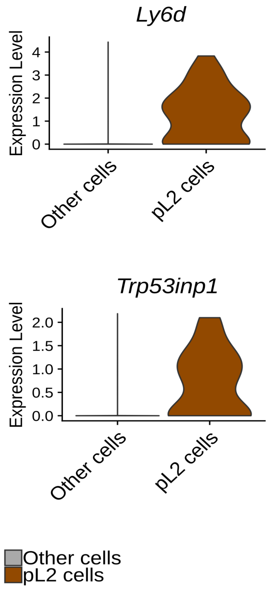

Supplemental Figure S3. Violin plots showing Ly6d and Trp53inp1 expression significantly up regulated in the pL2 cells cluster in comparison to the other cells (p-value < 0.05 & log fold change > 0.25).

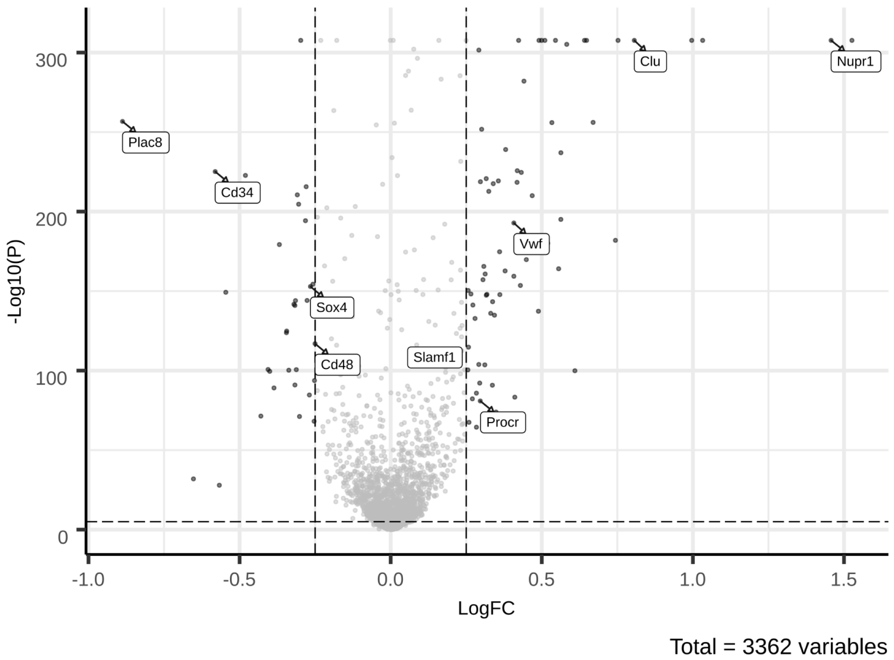

Supplemental Figure S4. Volcano plot of differential expression upon aging tested on all cells. Black dots indicate significant differentially expressed genes (DEGs; p-value < 0.05 and log fold change > 0.25). A total of 3362 genes were tested.

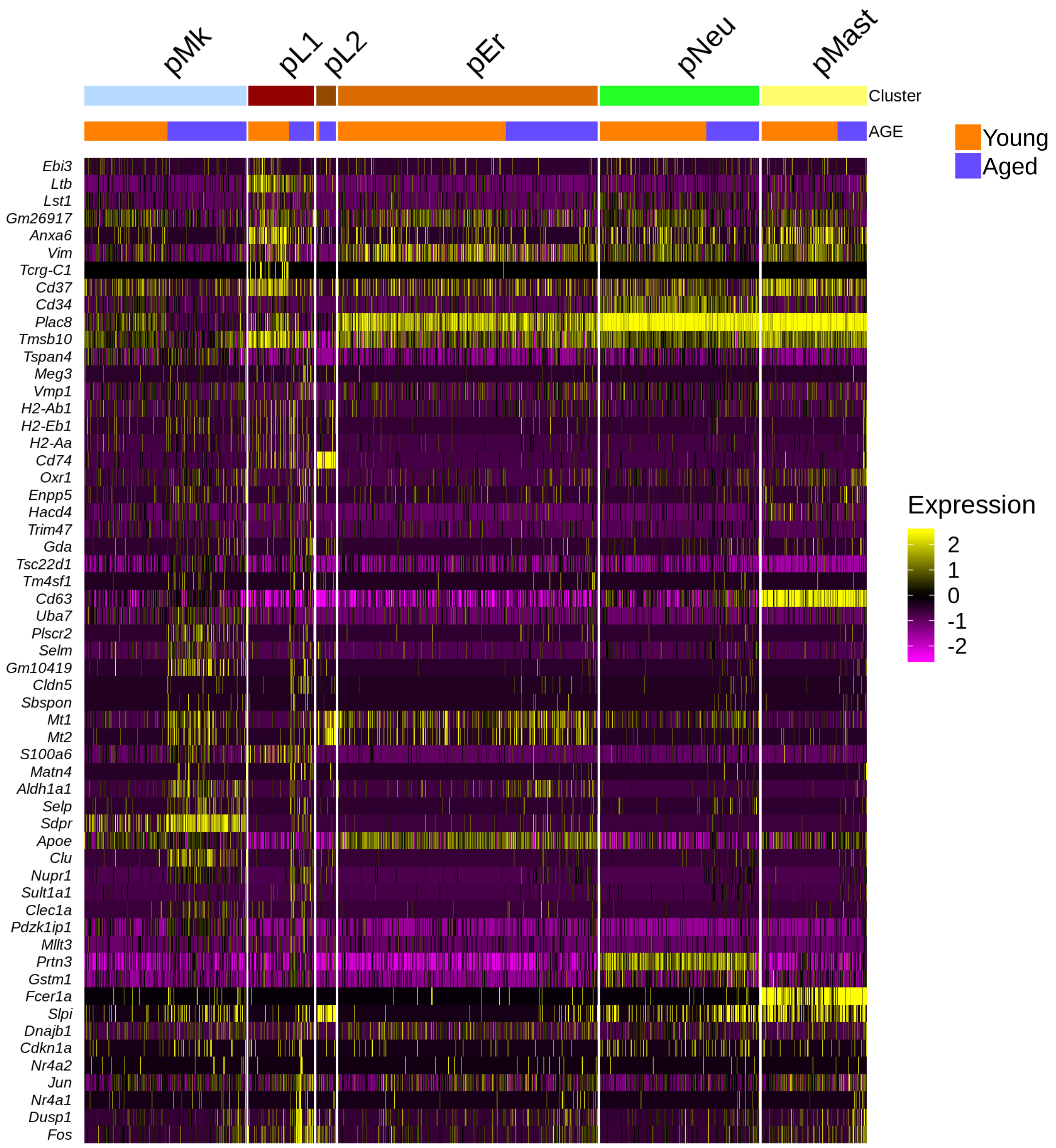

Supplemental Figure S5: Heatmap of the most significant DEGs upon aging (p-value < 0.05 and log fold change > 0.5 in at least one cluster) in the 6 lineage-primed clusters revealed by the Seurat analysis (Fig. 1b). Gene expression is standardised across the entire dataset.

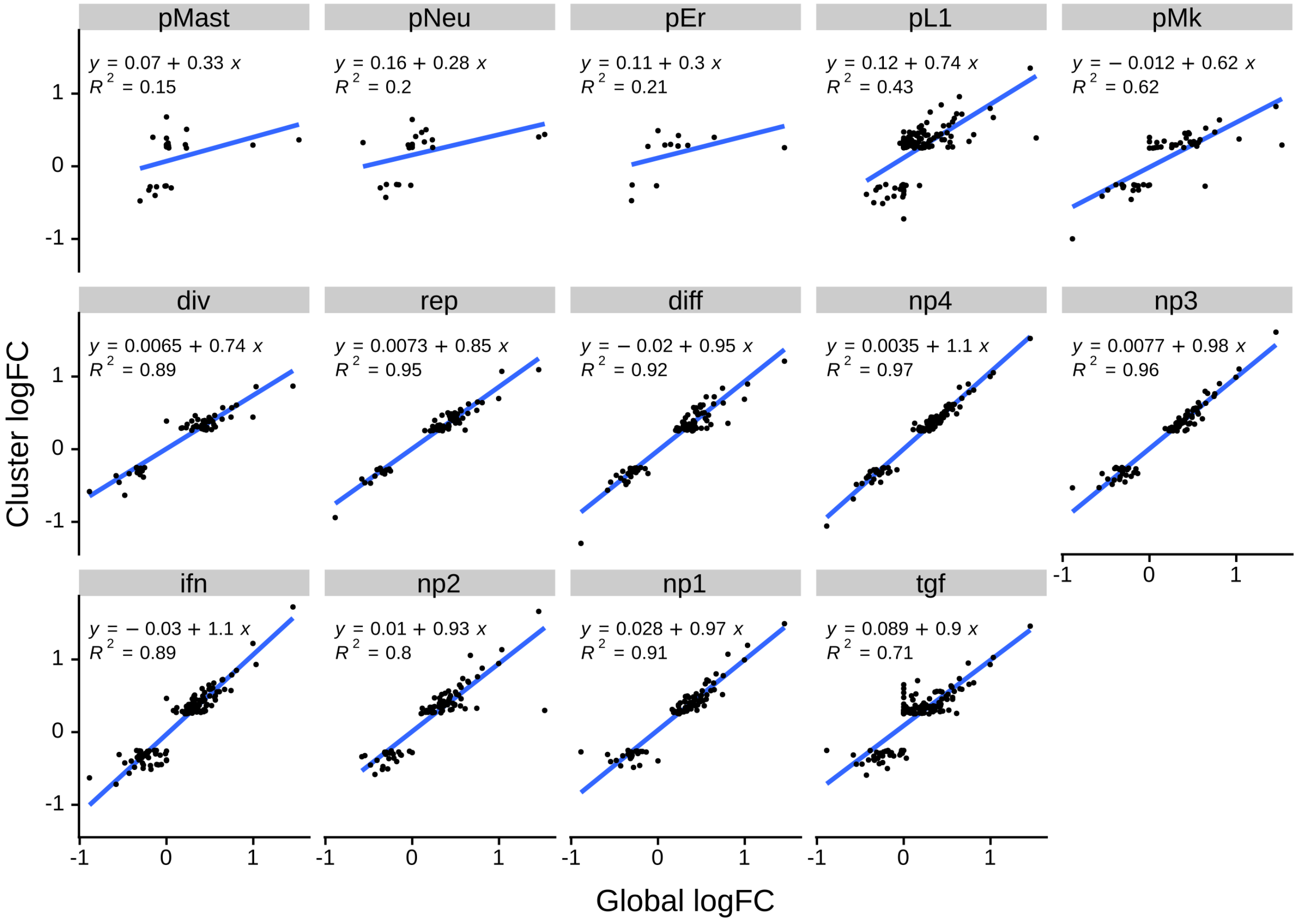

Supplemental Figure S6. **Comparison of cluster gene expression changes with global gene expression changes upon aging**. For each significant aging marker in a given cluster (p-value < 0.05 and log fold change > 0.25), its global log fold change (logFC; x-axis) is plotted with its cluster log fold change (logFC; y-axis). For each cluster, a regression line is drown in blue, formula is indicated at the left top corner with its square regression coefficient R^2.

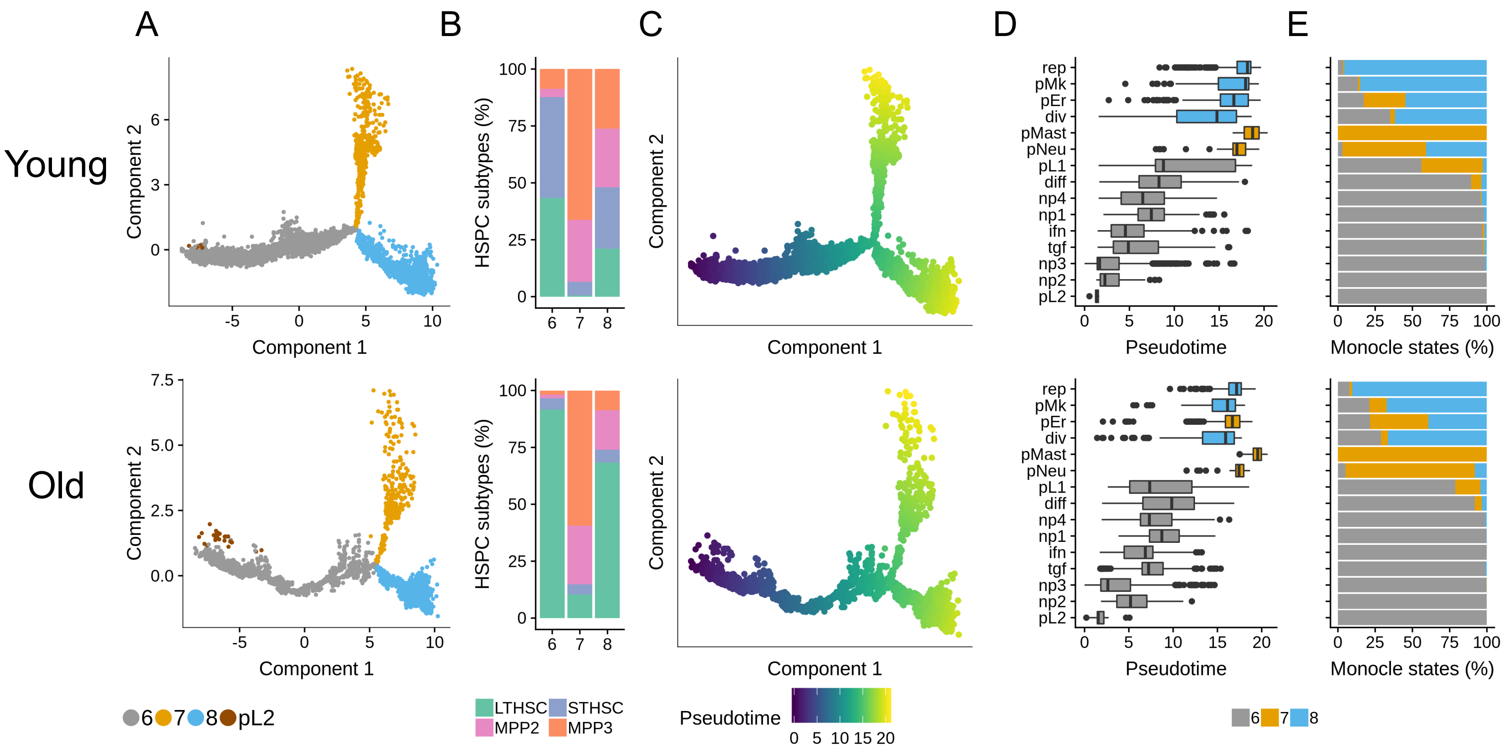

Supplemental Figure S7. (A) Monocle trajectories for young and aged HSPCs ordered separately. Cells are coloured according to their belonging to the three states (6 grey, 7 yellow, 8 blue) or to the pL2 cluster (brown). Both trajectories present a similar segregation between the lineage-primed HSPCs, with one bifurcation from LTHSC (state 6) towards Neu/Mast-primed (NeuMast) HSPCs (state 7) and Mk/Er-primed (MkEr) HSPCs (state 8). The bifurcation to lymphocyte fate was not retrieved, probably due to the reduction in pL1 cell number due to sample splitting. (B) Barplots representing the LTHSC, STHSC, MPP2 and MPP3 proportions in the three states. (C) Monocle trajectories of young and aged HSPCs coloured in accordance to their pseudotime values and representing their differentiation progression. (D) Repartition of the Seurat clusters along the pseudotime of young and aged HSPC trajectories. Box plots of pseudotime values are coloured according to the most represented state. (E) Repartition (in percentage) of the different states (6 to 8) of the trajectory for each Seurat cluster for young and aged HSPCs.

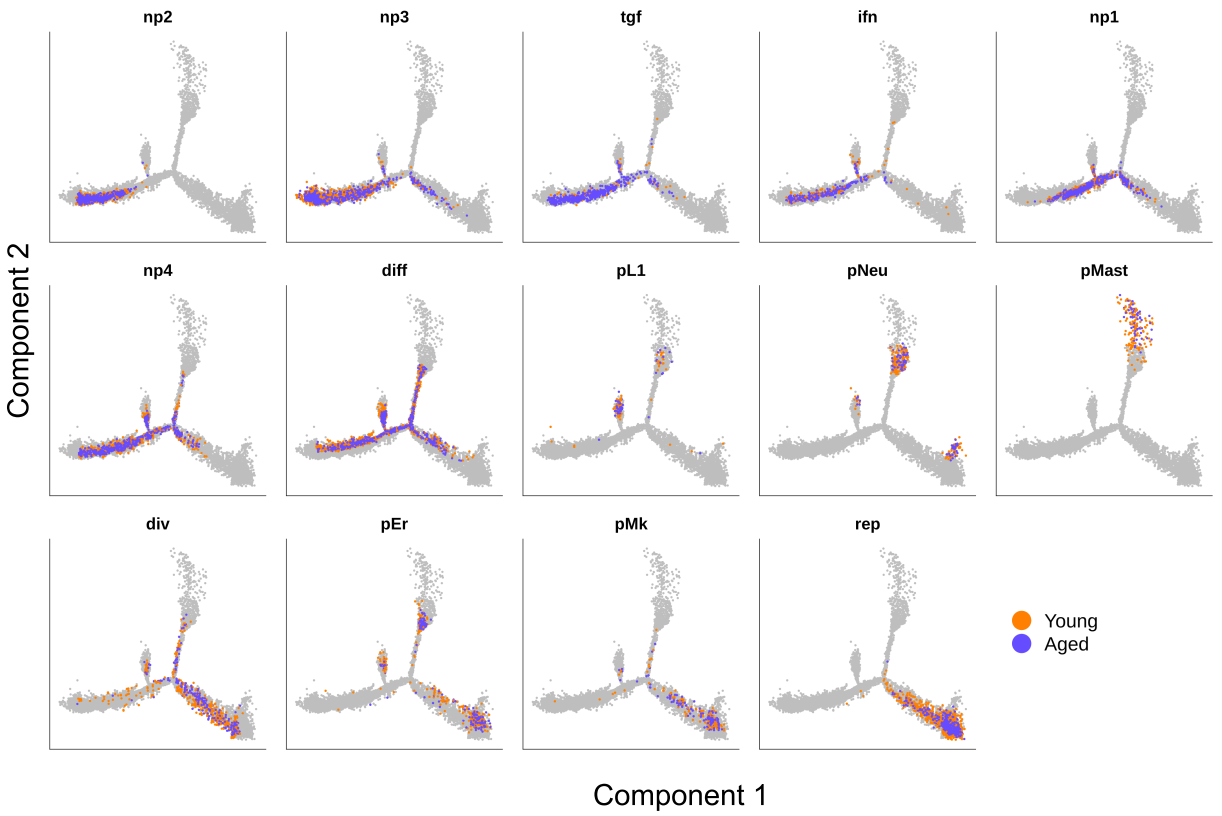

Supplemental Figure S8. Localization of the different Seurat clusters in Monocle trajectory. Cells belonging to a given cluster are coloured in orange for young and in purple for aged HSPCs.

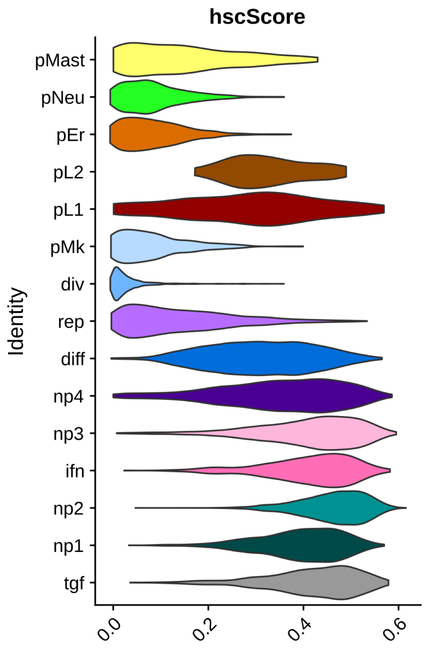

Supplemental Figure S9. **Analysis of the hscScore according to Seurat clusters.** Violin plots of hscScore distribution is presented in the 15 clusters.

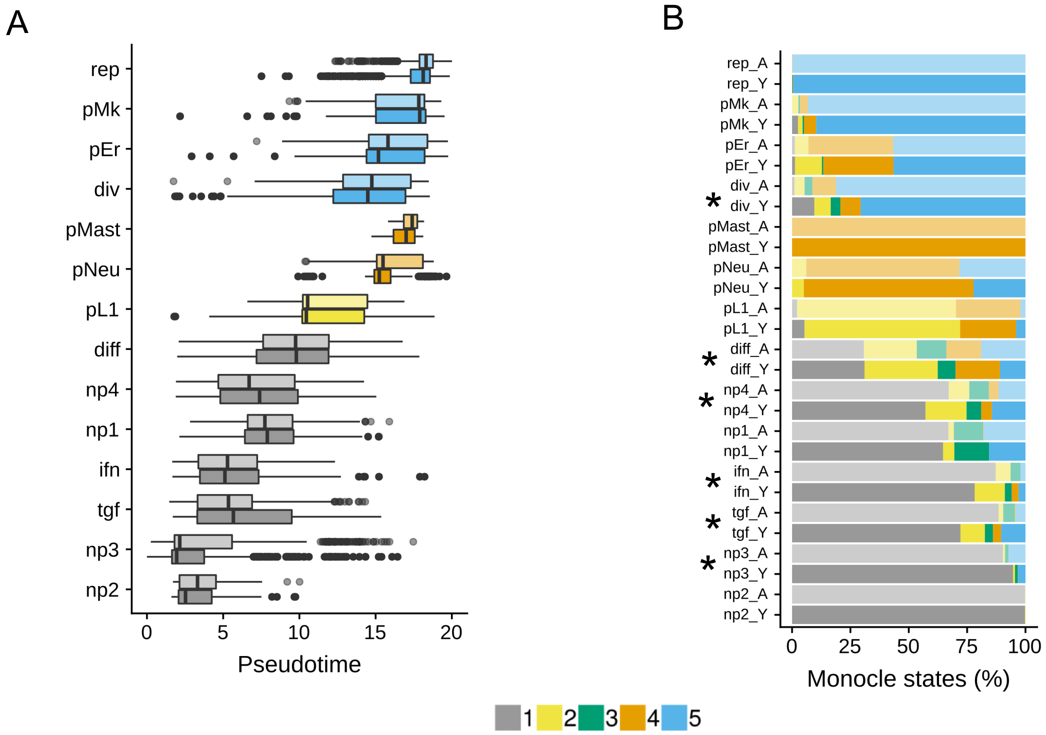

Supplemental Figure S10. **Repartition of young and old HSPCs in Monocle pseudotime and in states per Seurat cluster.** (A) Boxplots of Monocle pseudotime values of the young (dark) and aged (pale) cells from the different clusters obtained with Seurat (except pL2 cluster). Box plots showing medians are coloured according to the most represented state. (B) Comparison of Monocle state percentage in the different clusters between young (Y, dark colours) and aged (A, pale colours). Stars indicate a significant dependence between state repartition of the cells and age (p-value < 0.05 Fisher's Exact Test).

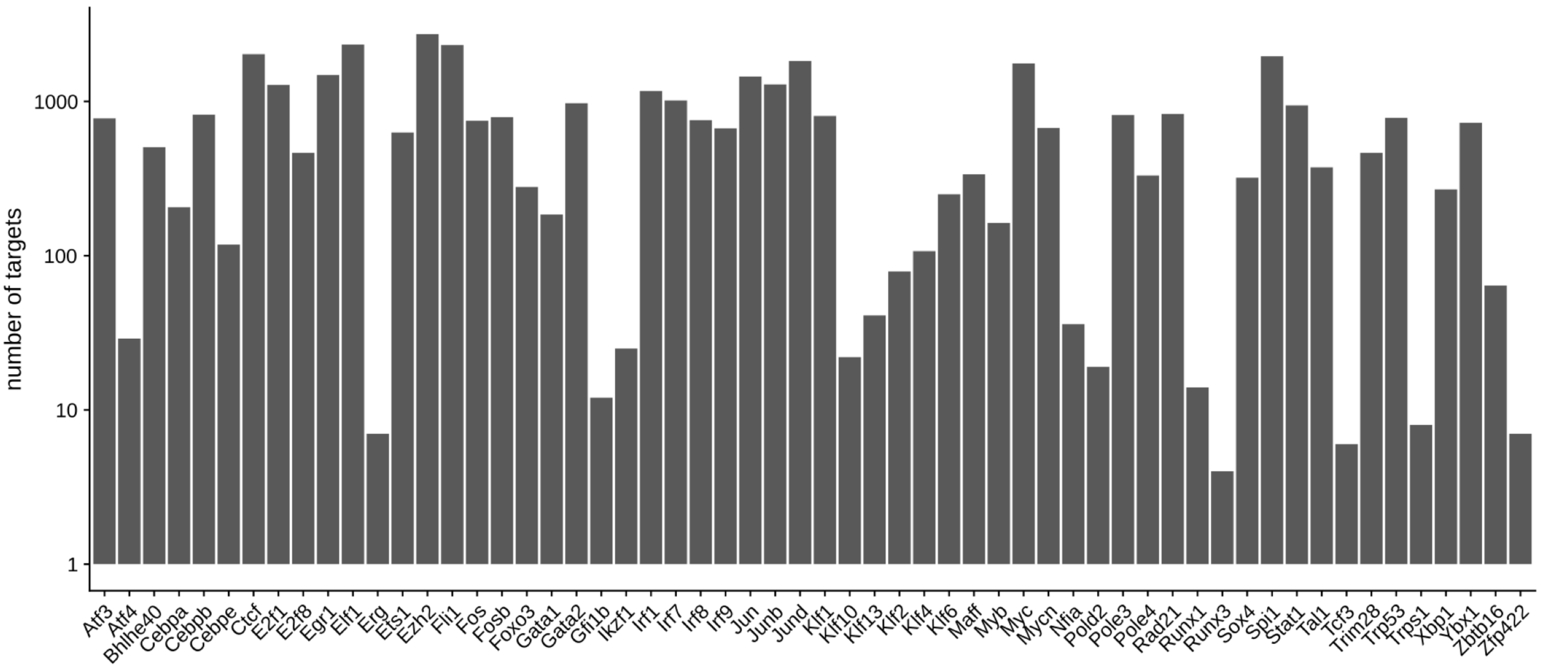

Supplemental Figure S11. Number of targets recovered for each regulon identified with scenic**.** Y axis is in log scale.

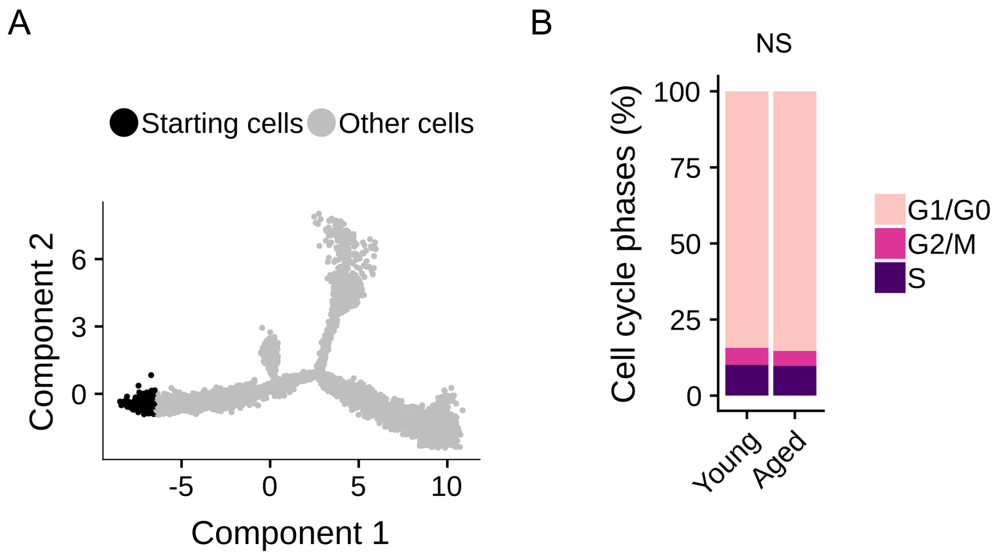

Supplemental Figure S12. **Young and aged HSPCs located at the very beginning of the trajectory cycled the same**. (A) Highlight in the trajectory of the starting cells (coloured in black, pseudotime < 2). (B) Cell cycle phase prediction of young and aged starting cells, highlighted in A. NS: no significant dependence between age and phase repartition (p-value > 0.3 Pearson's Chi-squared test).
